# Discerning dangerous gain of function: most gain of function (GoF) research does not involve infectious microbes

**DOI:** 10.3389/fbioe.2026.1818657

**Published:** 2026-06-19

**Authors:** Gene D. Godbold, C. Matt Sharkey, Bryan T. Gemler, Craig M. Bartling, F. Curt Hewitt, Joshua C. Gil

**Affiliations:** 1 Signature Science LLC, Charlottesville, VA, United States; 2 Center for AI, Security, and Technology, RAND Corporation, Santa Monica, CA, United States; 3 Battelle Memorial Institute, Columbus, OH, United States; 4 Signature Science LLC, Austin, TX, United States

**Keywords:** dangerous gain of function (DGoF), dual use research of concern (DURC), gain of function (GOF), microbial pathogenesis, sequence of concern (SoC)

## Abstract

The article reviews the use of the term “gain of function” (GoF) and “dangerous gain of function” (DGoF) in both the regulatory space relevant to the United States of America and the biomedical literature. Methods: We identified nearly 20,100 papers in PubMed which mentioned “gain of function” in the title or abstract. Through a series of machine and human evaluations, these were resolved to 145 that appeared to be dangerous gain of function (DGoF). In addition, we evaluated some papers that did not mention gain of function but involved expression of recombinant sequences of concern (SoCs) in heterologous microbes where the latter is endowed with pathogenic or toxic function as a result of the transfer. For 22 viral GoF papers, we discuss criteria leading to determinations of whether DGoF occurred with reference to the seven DURC criteria and discuss challenges in evaluating them. Principal Findings: We found that the overwhelming majority of the use of the “gain of function” term is not concerned with dangerous gain of function research. Less than 1% of the articles that use the GoF term discuss DGoF research. Over 40% of those are review or policy papers that do not deal with empirical research. Of the remaining experimental GoF articles, the majority involve antimicrobial resistance (AMR) in pathogens. In addition, it appears that most research that could be considered as DGoF according to the seven criteria for dual use research of concern (DURC) is not characterized by the authors as GoF. Conclusions: 1) most GoF research is not DGoF, 2) most DGoF is not referenced with the GoF term, and 3) discerning DGoF from GoF is not always straightforward but is helped by recognition rubrics, particularly the DURC criteria, as well as examples of SoCs with written functional definitions.

## Introduction

On 5 May 2025 the White House issued an executive order (EO), Improving the Safety and Security of Biological Research (EOP, 2025), which included a prohibition of federal funding for dangerous gain-of-function (DGoF) research on any infectious agent and or toxin as it could “lead to the proliferation of research on pathogens (and potential pathogens) in settings without adequate safeguards”. The research prohibited from being federally funded in the EO meet the criteria of seeking to achieve one of seven experimental outcomes that could enhance the ability of the agent to do harm and further must pose a risk of significant societal consequences. These criteria are non-trivial to apply to highly technical life sciences research proposals, and both extramural NIH-funded researchers and the NIH Office of Science Policy have reportedly had difficulty applying them in a uniform manner (Cohen and Kaiser, 2025; Gillum, 2025). Specifically, the application of the research oversight requirement or prohibition criteria to specific research proposals requires a working knowledge of pathogenesis in the microbe being studied–including details of the biological components being modified (often sequences of concern), technical experimental considerations, and the ability to anticipate the resulting biological properties of the modified agent and its altered activity in a natural host organism. A further complexity in the EO is that it calls for the development of a strategy to “govern, limit and track [DGoF] research across the United States that occurs without federal funding” and also asks for a legislative proposal where authorities are currently insufficient to prohibit, oversee, or otherwise govern DGoF research in the private sector. There is an urgent need to develop a comprehensive and uniform understanding of which research is covered by the federal funding prohibition in the EO and which may be covered in legal frameworks developed pursuant to it. The implementation of previous high consequence research oversight policies may indicate where challenges are likely to arise and may provide guideposts for what a new implementation mechanism should try to achieve.

The first research oversight policies that included the research outcomes in the DGoF definition were issued by the White House Office of Science and Technology Policy and NIH, in 2012 and 2014, respectively (United States Government, 2012; United States Government, 2014). The dual use research of concern (DURC) criteria of reasonably anticipated experimental effects, which are also applied in the newer DGoF policy, are:

- Enhances the harmful consequences of the agent or toxin;
- Disrupts immunity or the effectiveness of an immunization against the agent or toxin without clinical or agricultural justification;
- Confers to the agent or toxin resistance to clinically or agriculturally useful prophylactic or therapeutic interventions against that agent or toxin or facilitates their ability to evade detection methodologies;
- Increases the stability, transmissibility, or the ability to disseminate the agent or toxin;
- Alters the host range or tropism of the agent or toxin;
- Enhances the susceptibility of a host population to the agent or toxin; or
- Generates or reconstitutes an eradicated or extinct agent or toxin (i.e., rinderpest virus, 1918 influenza virus, or either variola major or minor viruses).

The 2012 and 2014 DURC policies required oversight of research in which these outcomes were anticipated if it involved one of 15 pathogens or toxins, all of which are also listed by the Federal Select Agent Program as biological select agents and toxins. So, the application of the criteria was limited to scientists used to doing biorisk assessments and to interacting with a governmental oversight regime. The expansion of oversight requirements for federally funded research, in the 2017 HHS Potential Pandemic Pathogen Care and Oversight (P3CO) to experiments reasonably anticipated to enhance the transmissibility or virulence of “any pathogen that is likely highly transmissible and likely capable of wide and uncontrollable spread in human populations; and … likely highly virulent and likely to cause significant morbidity and/or mortality in humans” posed a greater challenge for scientists and federal funding agencies (Department of Health and Human Services, 2017). The federal government faced public scrutiny over its application of the P3CO Framework (Inglesby and Lipsitch, 2020; Koblentz and Casagrande, 2023), with experts external to the federal government calling for clear lines to be drawn and transparently applied. Understanding the biochemical and immunological effects on the host of particular genetic elements that endow pathogens with harmful effects is important and helpful for the evaluation of research for the applicability of these policies, whose salient aspects are summarized in Table 1.

**TABLE 1:** Summary of criteria for oversight or prohibition of GoF/DGoF in the United States of America, 2012–present^1^.

Category	Term of effect	Category criteria
Dual Use Research of Concern (DURC)	2012–2025	DURC oversight requirements encompassed research, with 15 biological select agents and toxins, that was reasonably anticipated to result in one or more of seven experimental outcomes anticipated to increase the potential for harm from the agent
Category 1 (DURC)	2024–2025*	DURC oversight requirements encompassed research, with over 100 pathogens and toxins, that was reasonably anticipated to result in one or more of nine experimental outcomes anticipated to increase the potential for harm from the agent
Potential pandemic pathogen (PPP)	2017–2025	A pathogen that is both: 1) likely to be highly transmissible and likely to be capable of wide and uncontrollable spread in human populations; and 2) likely to be highly virulent and likely to cause significant morbidity and/or mortality in humans
Enhanced potential pandemic pathogen (ePPP)	2017–2025	An ePPP results from the enhancement of the transmissibility and/or virulence of a PPP. Naturally occurring pathogens that are circulating or that have been recovered from nature were excluded from this category, regardless of their pandemic potential
PPP	2024–2025	A pathogen that is likely capable of wide and uncontrollable spread in a human population and would likely cause moderate to severe disease and/or mortality in humans
Pathogen with enhanced pandemic potential (PEPP)	2024–2025	A PEPP results from the enhancement of the transmissibility, virulence, or immune evasion of a PPP in humans, the generation of an eradicated/extinct PPP (i.e., variola or 1918 influenza virus), or the generation of a PPP from a non-PPP.
Dangerous Gain of Function Research (DGoF)	2025 – Present	DGoF includes research with any pathogen or toxin that meets the 7 experimental categories established in the 2012 DURC policy and that could result in significant societal consequences

* *This category was pending implementation prior to rescission*.

Knowing the genome of a pathogen (e.g., a new Betacoronavirus isolated from bats) does not currently allow us to anticipate whether it is capable of causing a human pandemic. That said, the seven DURC anticipated experimental outcomes can, for the most part, be correlated with discrete genetic changes, including the insertion of ‘new’ genes from different organisms. The criteria for inclusion into the DGoF policy’s prohibition criteria include both the DURC experimental outcomes as well as the enhancement of transmissibility or pathogenicity criteria that were key to inclusion under the P3CO or PEPP policies, from 2017 or 2024, respectively. This paper is intended to show how understanding the capabilities of individual genes, with respect to pathogen-host biology during infection, can be applied to making determinations on the applicability of these oversight or prohibition policies.

This is particularly important for the life sciences research enterprise because, in the current environment, where there are uncertainties about how to apply the DGoF policy, it is likely scientists are hesitant about submitting critical research proposals aimed at understanding the mechanisms of transmissibility and virulence (Cohen and Kaiser, 2025; Gillum, 2025). So, we propose a framework in which the DGoF criteria can be understood through a better classification of the genes that can lead to DGoF enhancements. Through understanding how specific microbial genes, particularly SoCs and their functions, contribute to these enhancements, clearer boundaries may be established that can be uniformly and systematically applied by funding entities and understood by researchers, biosafety review committees, and research institutions alike so as to facilitate the safe and secure investigation of microbial pathogens.

## Use of the “gain of function” term in PubMed

The gain of function (GoF) designation describes the manifestation of one or more new functions in an organism as a result of natural or experimental genetic modification. The term is common in biomedical literature and has been used in the title or abstract of more than 20,099 publications (see Methods, below). Over 73% (14,750 abstracts) are concerned with gain of function mutations in (mostly) human sequences that concern oncology, neurobiology, developmental biology, immunology, or other human genetic diseases in which there is no mention of a microbe. We denoted those abstracts as “Non-DGoF Human” abstracts.

**FIGURE 1:**
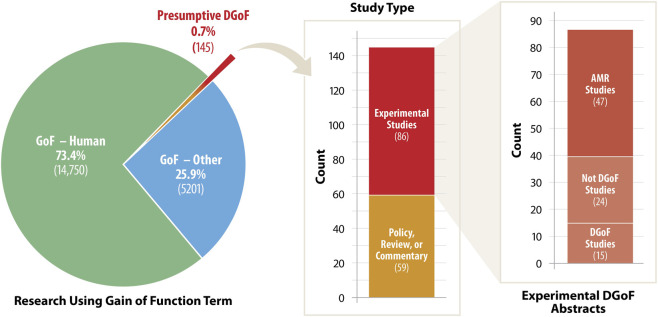
20099 articles from PubMed contain the term “gain of function” (GoF) in the title or abstract. This analysis assesses how many articles either mention or discuss dangerous gain of function (DGoF).

Of the residual 5,346 abstracts, 1,449 mention at least one microbial and one human term, 1,042 mention a microbial term only, and 2,855 did not match either a human or microbial term. The title and the AI-extracted terms of these abstracts were manually reviewed to provide a preliminary determination of DGoF followed by secondary review of the down-selected abstracts to confirm. Of those 5,346, just 145 appeared to be dangerous gain-of-function (DGoF) research according to the seven DURC criteria (Figure 1). Of those 145, 59 were either reviews, commentaries, or policy recommendation publications, while 86 featured experimental results. Of the 86 experimental papers, 47 were investigating GoF of antimicrobial resistance and the remaining 39 explored different facets of virulence.

Of the 47 GoF publications that introduce novel AMR empirical research, 27 characterize GoF resulting in antimicrobial resistance in species of the *Candida* (fungal) genus while 12 characterize GoF that produces antibiotic resistance in bacteria (*Acinetobacter baumannii*, *Enterococcus faecalis*, *Escherichia coli*, *Neisseria gonorrhoeae*, *Pseudomonas aeruginosa*, *Staphylococcus*, *Streptococcus*). There are similar papers in which antimicrobial resistance discovery is conducted for *Saccharomyces cerevisiae* (2 papers) and the protozoan parasite *Leishmania* (1 publication). These papers all use molecular mechanisms to understand genetic changes leading to gain of function experiments in natural microbial isolates. The pertinent observation is that there is no transfer of sequences from the microbes in which these were originally discovered to another microbe or to an artificial system in which human infection is likely.

Five of the empirical AMR papers are different. Two involve the treatment of SARS-CoV-2/Vesicular Stomatitis Virus (VSV) chimeras with antiviral drugs to generate resistant mutants. These are intended to discover and understand the molecular mechanisms of resistance (Costacurta et al., 2023; Rauch et al., 2024). One involves the engineering of azole resistance into *Candida albicans* to study the effects on the fitness and virulence of the fungal microbe in a mouse model of infection (Lohberger et al., 2014). Two used drug selection pressures on *Candida auris* (Li et al., 2024) or *S. cerevisiae* (Hnátová et al., 2003) to induce resistance so the mechanisms could then be explored and better understood.

There are 98 papers in which the term GoF is mentioned that do not concern antimicrobial resistance research, 59 are either reviews or commentary on GoF research. Thirty-nine involve experimental work. Of these, only 15 appear to involve actual DGoF research, falling into one or more DURC categories. One involves a fungal metalloprotease (Na Pombejra et al., 2017), two involve protein toxins (Fruchart-Gaillard et al., 2012; Krauson et al., 2012), five are viral papers, and seven involve bacteria (Stutzmann Meier et al., 2001; Cardwell and Martinez, 2009; Chang et al., 2011; Hair et al., 2013; Kao et al., 2017; Aghababa et al., 2022; Aspell et al., 2023). None of the bacterial SoCs are from select agents; they concern *Rickettsia conorii*, *Staphylococcus aureus*, *Streptococcus pyogenes*, or *Treponema pallidum*. Similarly, none of the viruses are select agents: *Hepacivirus hominis* (HCV) (Grobler et al., 2003), *Lentivirus simimdef*/*Lentivirus humimdef1* (SIV/HIV-1) (Sato et al., 2018; Nakano et al., 2020), *Rotavirus alphagastroenteritidis* (Wang et al., 2011), and *Orthoflavivirus japonicum* (JEV) (Fan et al., 2019).

This analysis indicates that the vast majority of the use of the “gain of function” term is not describing alterations of microbes pathogenic to humans in ways that make them more pathogenic. Because the work covered by “GoF” is so broad, as well as so generally innocuous, it seems a poor choice as a descriptor for research that is problematic because it poses a potential danger to human health. As we will show below, the researchers who investigate the properties of sequences encoded by pathogens and discover their capabilities by exploring if they can endow heterologous microbes with novel pathogenic function, do not generally use “GoF” to describe their work.

## Gain of function experiments in which a transferred SoC endows a heterologous microbe with pathogenic function

For various US government-funded projects having to do with sequence screening, we have been collecting and annotating the functions of SoCs since 2005. SoCs are sequences documented to endow a microbe with pathogenicity or toxicity. Documentation requires published empirical research of the sequence function with respect to host biology. While assembling datasets including thousands of SoCs for screening synthetic nucleic acids we found that very few SoCs had an empirically demonstrated capacity to endow a microbe from a *different* species—a heterologous microbe—with pathogenicity or toxicity. Instead, for the great majority of SoCs, researchers showed–using biochemistry, molecular genetics, and other techniques–that the sequences in question provided the original microbe with pathogenic or toxic capacities (Godbold et al., 2022). However, we have found 62 papers in which one or more sequences was investigated for pathogenicity in a different microbe. In these experiments, researchers demonstrate that the heterologous microbe is endowed with one or more pathogenic functions as a result of the expression of the sequence. Some of these sequences are discussed in the following section. A table with all of these sequences and a brief summary of the expression in the heterologous microbe and the results are provided in the Supplementary Material (Supplementary Tables 1). Only a dozen of these publications invoked the “gain of function” term in the title or abstract.

The functions these sequences provide to the heterologous microbe that identify them as SoCs include one or more of the following: the ability to (1) damage host cells, tissues, or organs; (2) subvert host innate immune signaling (e.g., via interferons, MAP kinases, NF-κB); (3) counter host innate immune effectors (e.g., antimicrobial peptides, complement, cytokines, immunoglobulins, phagocytes, reactive oxygen species); (5) enable dissemination through host defenses; (6) invade host cells; (7) adhere to host cells and tissues; (8) otherwise hijack host cell biology including interfering with or commandeering host autophagy, cell cycle, cytoskeleton, endomembrane dynamics, regulated cell death, small GTPases, transcription, translation, or ubiquitination. SoCs enable a microbe to cause disease by making hosts susceptible to colonization (Gemler et al., 2022; Godbold et al., 2022). While these sequences are necessary for pathogenesis, it is impossible to predict how this would manifest in the encoding organism in the absence of experiments that also involve a host. The presence of one or more sequences that possess these functions in a pathogen genome do not enable the prediction of organismal, epidemiological, or public health-level risk.

### SoCs demonstrating adherence in heterologous microbes: the “floor” of pathogenesis

There are numerous experiments in which the transfer of a sequence has been shown to endow a microbe, usually a bacterium, with the ability to adhere to a biological substrate. These experiments are common enough that specialized weakly- or non-adherent bacteria have been specially developed to enable them. The work typically involves expressing a suspected adhesin from a particular bacterial pathogen in a non-pathogenic bacterium from the same genus or else in a non-pathogenic laboratory strain of *E. coli*. The resulting recombinant bacterium is then analyzed using a mammalian cell line (immortalized epithelial, endothelial, or other cells) or receptor binding assay. We consider adherence to be the “floor” of pathogenic function. While it is often a necessary precondition for other deleterious capacities, we have not seen any examples in which a sequence that *only* has the adhesin function, upon transfer to a heterologous, non-pathogenic microbe, endowed it with the ability to harm a host. This does not fulfill any of the DURC criteria.

Many adhesins bind fibronectin, an important glycoprotein forming the extracellular matrix (ECM). This can sometimes enable invasion into host cells. For example, fibronectin-binding proteins from *Staphylococcus aureus* and pathogenic *Streptococcus agalactiae*, when expressed in nonadherent strains, bind fibronectin and invade epithelial and/or endothelial cells (Schubert et al., 2004; Mu et al., 2014; Lyon et al., 2023). In addition to fibronectin-binding proteins, other adhesins bind other superficial components of host cells. Surface antigens (e.g., BspA and BspC) from Group B Streptococcal species such as *Streptococcus agalactiae* (GBS), when expressed on the surface of non-adherent *Lactococcus lactis* endows the bacteria with adherence to scavenger receptor gp340 (Rego et al., 2016; Deng et al., 2019). Surface cell antigens (Sca0, Sca1, Sca2, and Sca5) from pathogenic *Rickettsia* species, when expressed in *E. coli* mediate adherence to primate epithelial cells (Uchiyama et al., 2006; Cardwell and Martinez, 2009; Riley et al., 2010; Hillman et al., 2013).

Asc10 (PrgB) from *Enterococcus faecalis*, when expressed in *Lactococcus lactis* and *Streptococcus gordonii* contributes to fibrin adhesion (Hirt et al., 2000; Holmes et al., 2001).

Other adhesins bind immunoglobulin receptors for attachment. Pneumococcal surface protein C (PspC) from *Streptococcus pneumoniae*, when expressed in *Lactococcus lactis* mediates adherence and invasion (Asmat et al., 2012). Similarly, *Leptospira interrogans* immunoglobulin-like protein A (LigA) and LigB, when expressed in saprophytic *Leptospira biflexa*, significantly enhances the bacterium’s ability to adhere *in vitro* to cultured cells and ECM components (Figueira et al., 2011).

Internalin A (InlA) from *Listeria monocytogenes* (Guimarães et al., 2005), *Listeria* adhesion protein B (LapB) from *Listeria monocytogenes* (Reis et al., 2010), *Haemophilus influenzae* high molecular weight proteins (HMW1 and HMW2) (St Geme et al., 1993), and Opacity-associated protein A (OapA) (Prasadarao et al., 1999), adhesion and penetration protein (App) (Serruto et al., 2003), meningococcal serine protease A (MspA) and from *Neisseria meningitidis* (Turner et al., 2006), major outer membrane protein (MOMP) from *Legionella pneumophila* (Krinos et al., 1999), pallilysin (Tp0751) and surface lipoprotein Tp0954 from *Treponema pallidum* (Kao et al., 2017; Primus et al., 2020). Others include attachment invasion locus protein (Ail) (Bliska and Falkow, 1992; Bartra et al., 2008), autotransporter protein K and V (YapK/V) from *Yersinia* species (Nair et al., 2015), and trimeric autotransporters from *Brucella suis* (Ruiz-Ranwez et al., 2013), *Burkholderia pseudomallei* (Lafontaine et al., 2014), *Bartonella henselae* (Schmidgen et al., 2014), *Burkholderia mallei* (and *B. pseudomallei*) (Balder et al., 2010), *Neisseria meningitidis* (Capecchi et al., 2005; Scarselli et al., 2006), and *Bartonella quintana* (Zhang et al., 2004).

A few “gain of adherence” experiments have been conducted in eukaryotes.

- Agglutinin-like sequence three protein (Als3p) from *Candida albicans*, when expressed in *Candida glabrata* (a fungi with low virulence in mice), has enhanced adherence to and invasion of human brain microvascular endothelial cells *in vitro* (Fu et al., 2013). Als3p is not just an adhesin but also an invasin and this experiment could represent DGoF, DURC criteria (A), though the authors do not invoke the GoF term.
- Mpr1 from *Cryptococcus neoformans*, when expressed in *Saccharomyces cerevisiae* allows the yeast cells to migrate across human brain microvascular endothelial cells in an *in vitro* static model of the blood-brain barrier (Na Pombejra et al., 2017). This is another example of DGoF.
- Penetrin from *Trypanosoma cruzi*, when expressed in *E. coli*, enables bacterial adhesion to collagen, heparin, and heparan sulfate (Ortega-Barria and Pereira, 1991).

There are thousands of adhesins in biology. Adherence forms the floor of a hierarchy of pathogenic function. Although adherence to a host cell or substrate cannot, by itself, make a non-pathogen pathogenic, adherence is often a necessary precondition for further deleterious interaction that enables a pathogenic process. And adhesins can have functions beyond adherence that are pathogenic.

### *M. tuberculosis* SoCs: “dangerous” GoF research for rational reasons

A good reason to express and study a sequence from a controlled microbe, in which BSL-3/BSL-4 procedures must be followed, in a related non-controlled microbe is to avoid the onerous (= expensive and time-consuming) procedures associated with the more stringent research environment. For research involving *M. tuberculosis*, not only is work more difficult because it requires BSL-3 conditions, but the pathogenic *Mycobacteria* species *avium*, *leprae*, and *tuberculosis* grow very slowly. *M. tuberculosis* has a doubling time of 18–54 h. In contrast, the nonpathogenic but genetically similar *Mycolicibacterium smegmatis* has a doubling time of just 2–5 h (Gill et al., 2009). Expression systems developed over the last 30 years make *M. smegmatis* tractable for genetic manipulation. This allows the function of *M. tuberculosis* sequences and processes to be studied in a microbe in which a colony appears on a plate in 3 days, not 3 weeks (Sparks et al., 2023).

Dozens of proteins from *M. tuberculosis* have been demonstrated to endow *M. smegmatis* with pathogenic functions. Many influence the programmed cell death (PCD) of host macrophages, others enable host cellular functions to be hijacked for the benefit of bacteria. Some enable the subversion of host innate immune pathways. These SoCs are briefly summarized below:

*M*. *tuberculosis* SoCs that inhibit macrophage PCD: NuoG (in *M. kansasii*) (Velmurugan et al., 2007), PE-PGRS18 (Yang W. et al., 2017), PE-PGRS38 (Ullah et al., 2024), PE_PGRS41 (Deng et al., 2017), PE31 (Ali et al., 2020), PE-PGRS62 (Long et al., 2019), Rv0426c (Ruan et al., 2020).

*M*. *tuberculosis* SoCs that promote macrophage PCD: Esx (Mohanty et al., 2016), LpqT (Su et al., 2016), Mpt83 (Wang et al., 2017), PE13 (Li et al., 2016), PE-PGRS19 (Qian et al., 2022), PPE27 (Yang G. et al., 2017), PPE32 (Deng et al., 2016), Rv0177 (Yan et al., 2019), Rv1509 (Manjunath et al., 2024), Rv3717 (Gong et al., 2020).

### *M. tuberculosis* SoCs that hijack host cellular functions

1. Transcription: Histone acetyltransferase Rv3423.1 manipulates the expression of host macrophage genes involved in anti-inflammatory responses (Jose et al., 2016) The decrease in MHC class I on the surface of macrophages infected with *M. marinarum* expressing PPE38 is thought to occur at the level of transcription (Meng et al., 2017).
2. Ubiquitination: *M. smegmatis* expressing PE-PGRS38 infecting macrophages inhibit deubiquitination of macrophage TRAF6 by USP7 (also known as herpesvirus-associated ubiquitin-specific protease/HAUSP) by direct interaction with USP7. This decrease in TRAF6 ubiquitination resulted in a decrease in host proinflammatory cytokine secretion that increased the intracellular burden of bacteria (Kim et al., 2022).
3. Cytoskeleton: PE18 alone, and in combination with PPE26, inhibits actin-mediated cytoskeletal changes that slow the maturation of phagosomes, thereby improving bacterial survival. *M. smegmatis* expressing either of these proteins suppressed the major regulators of actin polymerization and filamentation (host N-WASP and ARP2) in infected macrophages. Rac1 expression was also suppressed and profilin downregulated. The most profound effects were seen in *M. smegmatis* expressing both PE18 and PPE26 (Ehtram et al., 2025).
4. Endomembrane Dynamics:
  - PE_PGRS30 is required to block maturation of the phagosome. Expression of PE_PGRS30 in *M. smegmatis* allows the normally non-pathogenic bacterium to cause extensive cell death in a tissue culture monolayer of J774 murine macrophage-like cells. In contrast, wild-type *M. smegmatis* is rapidly destroyed by the cells (Iantomasi et al., 2012).
  - Rv3091 is an extracellular patatin-like phospholipase of *Mycobacterium tuberculosis.* When heterologously expressed in *M. smegmatis*, it prolongs the intracellular survival of the bacterium in murine peritoneal macrophages by allowing escape from the phagosome (Cui et al., 2020).
  - When *M. smegmatis* expressing the cell wall protein PE_PGRS62 infects murine macrophages, it induces an arrest in the host phagosome maturation pathway, acquiring the host small GTPase Rab5 but not Rab7 or LAMP-1 (Thi et al., 2013). When a macrophage cell line is infected with M. smegmatis expressing PE_PGRS62, host phagolysosome fusion is disrupted and bacterial survival is enhanced (Long et al., 2019).
  - When *M. smegmatis* expressing Rv1509, a putative DNA methyltransferase of 293 residues, infects RAW264.7 macrophages, it augments bacterial survival compared to cells expressing vector alone. This is the result of a suppression of both phagolysosomal maturation and nitric oxide levels inside cells (Manjunath et al., 2024).
5. Autophagy: Macrophages infected with *M. smegmatis* expressing PE_PGRS41 suppress autophagy through interference with host ATG-8 (Deng et al., 2017).

### *M. tuberculosis* SoCs that subvert cellular innate immune signaling

1. *Mycolicibacterium smegmatis* expressing the lipoprotein LpqT survive better in macrophages derived from murine bone marrow when compared to bacteria that lack the gene. LpqT suppresses induction of NF-κB and MAPK phosphorylation (Li et al., 2018). LpqT inhibits host MHC-II expression and processing of soluble antigens induced by interferon-γ. These deficiencies are caused by LpqT binding to TLR2 and the subsequent signaling through MAPK (Su et al., 2016).
2. *M. smegmatis* expressing PE_PGRS38, a protein of 532 residues found in the cell wall, perturbs the secretion of proinflammatory cytokines during infection of RAW264.7 macrophages. Expression of TLR4, IL-1β, IL-6, TNF, MYD88, and NF-κB are decreased in macrophages infected with bacteria expressing PE_PGRS38 while anti-inflammatory cytokine IL-10 is increased when compared to those expressing vector alone (Ullah et al., 2024).
3. *M. smegmatis* expressing recombinant Rv0927c and used to infect RAW264.7 murine macrophages demonstrated inhibited proinflammatory cytokine expression compared to bacteria expressing vector alone. Expression of recombinant Rv0927c in HEK293 cells was sufficient to inhibit the translocation of NFκB p65 subunit following stimulation with lipopolysaccharide. Rv0927c suppressed NFκB pathways by downregulating phosphorylation levels of IκBα (Xia et al., 2021).
4. Macrophages infected with *M. smegmatis* expressing the *M. tuberculosis* protein PE_PGRS41 decrease host pro-inflammatory cytokines IL-1β, IL-6, and TNF and are also less able to respond to activation by bacterial lipopolysaccharide when compared to macrophages infected with bacteria expressing just the vector (Deng et al., 2017).
5. When PE15 is expressed in the nonpathogenic *M. smegmatis* and used to infect cultured macrophages, the bacteria exhibit improved survival, decreased iNOS production, and activate host MAP kinases leading to production of the anti-inflammatory cytokine IL-10 (Tiwari et al., 2012).
6. THP-1 macrophages infected with *M. smegmatis* expressing PE31 suppress expression of pro-inflammatory cytokines IL-12 p40 and IL-6 and boost production of anti-inflammatory cytokine IL-10. The infected macrophages also upregulate expression of the host interferon-stimulated GTPase guanylate-binding protein-1 (GBP-1) when compared to macrophage infected with bacteria only expressing control vector. This occurs through regulation of the NFκB pathway (Ali et al., 2020).
7. PPE2 is a secreted protein with a nuclear localization sequence and a leucine zipper motif at its C terminus. When ectopically expressed in activated RAW264.7 macrophages, it moves into the host nucleus and inhibits nitric oxide (NO) production by transcriptional repression, binding to the iNOS promoter. When PPE2 is expressed in *M. smegmatis*, it improves the survival of bacteria inside infected macrophages when compared to wild-type (Deng et al., 2016). PPE2 interacts with p67(phox) to inhibit ROS production (Srivastava et al., 2019).
8. When PE5 or PE15 or PE-PGRS62 are expressed in *M. smegmatis* and used to infect cultured macrophages, the bacteria downregulate expression of host iNOS and trigger activation of the host MAP kinases leading to production of the anti-inflammatory cytokine interleukin-10 (Tiwari et al., 2012; Thi et al., 2013).
9. When the cell wall protein PPE11 was overexpressed in *M. smegmatis*, it improved the viability of the bacterium in the presence of lysozyme, hydrogen peroxide and acid stress and improved the early survival within macrophages (Peng et al., 2019).

### SoCs that enable heterologous microbes to counter host innate immune effectors and suppress innate immune signaling

We hypothesize that the SoCs that make the greatest difference in pathogenesis are those that target the innate immune system of the hosts that their encoding microbes infect. We divide these into SoCs that counter host effectors (e.g., immunoglobulins, complement, reactive oxygen species, antimicrobial peptides, and cellular effectors such as macrophages, neutrophils, and natural killer cells) and those that suppress host innate immune signaling. We think the latter are probably more important since their inhibition can attenuate a host immune response, allowing the microbe to gain a temporal window in which host resistance is diminished. Countering effectors after the immune system is already mobilizing for danger, while it certainly helps to preserve the pathogen, is fighting a rear-guard action. Countering existing effectors is likely to provide less time for replication than blunting signaling to combat immune mobilization. *Streptococcus pyogenes* may be taking a hybrid approach with the expression of SpyCEP. This secreted protease cleaves host interleukin-8, a signaling cytokine produced by host cells local to the infection. If there are enough bacteria expressing and secreting the enzyme, it can dysregulate the localization of extravasating neutrophils to the site of infection such that expression of SpyCEP in a non-pathogenic bacterium, *Lactococcus lactis*, can turn it into a conditionally lethal pathogen (Kurupati et al., 2010).

### SoCs suppressing innate immune signaling when expressed in heterologous microbes

1. IcaA from *Coxiella burnetii* endow *Legionella pneumophila* with the ability to suppress the activation of host macrophage inflammasomes. IcaA inhibits inflammasome activation and subsequent caspase-1 activation in host macrophages by inhibiting host caspase-11-activation of the inflammasome somewhere upstream of caspase-11 (Cunha et al., 2015).
2. A mutant *Citrobacter rodentium* that expressed both NleH1 and NleH2 of *E. coli* was hypervirulent in a mouse model of infection. The infection was controlled by treating the mice with the anti-NF-κB peptide inhibitor SN-50 (Feuerbacher and Hardwidge, 2014). NleH1 and NleH2 repress the NF-κB pathway downstream of IKK and upstream of p65. They inhibit ubiquitination of the phosphorylated inhibitor of NF-κBɑ and dampen inflammation in the colon of EPEC-infected mice and promote bacterial colonization (Royan et al., 2010).
3. Overexpression of EHEC NleL in *C. rodentium* suppresses the ability of the mutant bacterium to attach to HeLa cells and form actin pedestals (Sheng et al., 2017). The T3SS effector NleL is a catalytic E3 ligase that has functional similarities to HECT E3 ligases and is involved in pedestal formation in attaching and effacing enteric bacteria (Piscatelli et al., 2011). NleL interacts directly with human JNK *in vitro* and *in vivo*. NleL promotes both the mono- and poly-ubiquitination of JNK1 at different residues and these modifications abolish JNK1 phosphorylation. JNK2 and JNK3 are similarly targeted. This NleL-mediated ubiquitination of host JNK proteins on Lys68 suppresses the interaction between JNK and host MKK7, thereby impairing JNK/AP-1 signaling (Sheng et al., 2017).

### SoCs countering host immune effectors when expressed in heterologous microbes

1. pH6 antigen from *Yersinia pestis*, when expressed in *E. coli*, reduces phagocytosis by murine macrophages (Huang and Lindler, 2004).
2. Expression of streptococcal SpnA by the non-pathogenic *Lactococcus lactis* gives this bacterium the ability to degrade extracellular DNA, escape NETs and increase survival time in both whole blood killing assays and neutrophil killing assays (Chang et al., 2011).
3. YadA from *Yersinia pseudotuberculosis*, when expressed in *E. coli*, promoted resistance to human serum comparable to those of *Y. enterocolitica* (Heise and Dersch, 2006).
4. Expression of Ail from *Yersinia enterocolitica* in *E. coli* increased serum resistance by 100-fold (Bliska and Falkow, 1992). Expression of Ail from *Y. pestis* in *E. coli* also allows the recombinant bacteria to resist complement-mediated killing (Bartra et al., 2008).
5. When LigA from *Leptospira interrogans* is expressed in a saprophytic *L. biflexa*, it confers enhanced survival to in human serum, with the deposition of membrane attack complex intermediate between *L. interrogans* and unmodified *L. biflexa* (Castiblanco-Valencia et al., 2016).
6. NalP from *Neisseria meningitidis*, when expressed in *E. coli* can cleave human complement component C3 (Del Tordello et al., 2014).
7. Expression of SdrE from *S. aureus* on the surface of *L. lactis* allows it to bind four-fold more host complement factor H than the wild type bacterium. *L. lactis* expressing SdrE produces less C5a and yielded less C3-fragment deposition than wild type (Sharp et al., 2012).
8. When expressed in the outer membrane of serum-sensitive *E. coli*, Adr1 from *Rickettsia conorii* interacts with human vitronectin and mediates resistance to serum killing (Riley et al., 2014).
9. When expressed in *Borrelia burgdorferi*, BhpA from *B. hermsii* confers greater resistance to oxidative stress and destruction by neutrophils (Guyard et al., 2006).
10. When expressed in *E. coli* that are susceptible to peroxide killing in the stationary phase because they lack superoxide dismutase, SodC from *C. burnetii* restores resistance to hydrogen peroxide (Brennan et al., 2015).
11. SpyCEP from *S. pyogenes* cuts and inactivates the human chemokine IL-8 and other human CXC chemokines that contain the ELR motif.(Hynes and Sloan, 2016). When expressed in the cheese-making firmicute *L. lactis*, it renders the bacterium pathogenic in mice (Kurupati et al., 2010).
12. When PI-PLC from *Bacillus anthracis* is expressed in *L. monocytogenes*, it is active toward GPI-anchored host (mouse) proteins (Wei et al., 2005). PI-PLC decreases host dendritic cell function and T cell responses, possibly by cleaving GPI-anchored proteins that are important for the toll-like receptor-mediated activation (Zenewicz et al., 2005).
13. Recombinant anthrolysin O from *B. anthracis*, when expressed in *E. coli* can lyse red blood cells and is lethal to a variety of different white blood cells including: human primary PMNs, primary monocytes, primary monocyte derived macrophages (MDMs), lymphocytes, THP-1 monocytic human cell lines, ME180, Detroit 562, and A449 cells.(Mosser and Rest, 2006). Introduction of the gene for ALO gene into *B. subtilis* results in the production of functional ALO and the ability of this bacterium to escape killing by cultured macrophages by inhibiting the bactericidal activities of these cells (Heffernan et al., 2007).

## Viral dangerous gain of function determinations

Determining whether a viral GoF paper involves DGoF can be difficult. To illustrate these difficulties, we chose three science professionals, each with at least 15 years’ experience in biodefense and/or virology (after graduate work) to evaluate a tranche of what appeared to be viral GoF papers in which a sequence of concern was transferred from one virus to another of a different species. Evaluator one was the most experienced and wrote an initial evaluation for the entries. Evaluators two and three then indicated agreement (or not) and added their comments. They agreed unanimously that nine of the 22 papers met the criteria for DGoF and that six did not. They split on seven papers. A brief description of each paper and the summarized comments of Evaluator one are given below in Table 2. The complete discussion of the three evaluators are provided in the Supplementary Material (Viral_DGoF_Discussion_Supplemental).

**TABLE 2:** Summary of expert review of 22 viral GoF studies.

Description	DGoF: not DGoF	Excerpted comments from evaluator 1
Influenza virus NS1 transferred to Newcastle Disease Virus (Fernandez-Sesma et al., 2006)	3:0	It should have been clear to the researchers that the experimental approach would result in a virus with enhancements to DURC categories (F) … and (A)
VP35 from Ebolavirus expressed in Newcastle Disease Virus (Leung et al., 2011)	3:0	[A]dding VP35 from Ebola virus to either NCDV or herpes simplex virus is reasonably anticipated to meet DURC category (F) … and (A)
E3L-like protein from Orf virus restores IFN resistance to vaccinia virus (Myskiw et al., 2011)	3:0	E3 proteins could inhibit PKR and IFN … adding them to E3L-deletion vaccinia was an experiment that could be reasonably anticipated to meet DURC (F) … and (A)
VP4 of Rhesus rotavirus (Wang et al., 2011)	3:0	The transfer of genes from rhesus rotavirus (RRV), which causes disease in humans, to the TUCH strain of rotavirus … in a manner that increases the pathogenicity of TUCH in an animal model of infection seems to meet DURC criteria (A)
Alphavirus eastern/EEV and the E2 glycoprotein (Gardner et al., 2013)	3:0	[S]ubstitutions intended to modify the interaction with heparan sulfate - their anticipated results would fall under DURC (A), enhancing the harmful consequences of the eastern equine encephalitis virus
NS2 from RSV expressed in parainfluenza virus 3 (Liesman et al., 2014)	3:0	[A]dd [NS2 protein] to another respiratory virus, parainfluenza virus (PIV), to see if it would make PIV more harmful to airway epithelia. The experimental aim satisfies DURC criteria (A)
Adapting SIV to pandemic HIV-1 using humanized mouse model (Sato et al., 2018)	3:0	[T]he introduction of mutations from human immunodeficiency virus (HIV) strain M to determine if they expand the host range and replication efficiency seem to meet DURC criteria (A) … and (E)
NS2B/NS3 mutations enhance the infectivity of Japanese encephalitis virus (Fan et al., 2019)	3:0	[I]ntroduction of mutations that further increase the replication efficiency of the GII/GI chimera seems to meet DURC criteria (A)
Role of gorilla APOBEC3G in shaping lentivirus evolution including human transmission (Nakano et al., 2020)	3:0	The generation of a mutant SIVcpzPtt that replicated in human cell lines meets DURC criterion (E)
Tat variants in HIV-1 (Verhoef and Berkhout, 1999)	0:3	The identification of Y47N as increasing Tat’s potentiation of reverse transcription by 200% but this does not actually benefit viral replication as shown in Fig. 7
Henipaviruses Glycoprotein shuffling (Bossart et al., 2002)	0:3	They use vaccinia virus as a transduction mechanism to get expression of henipavirus envelope glycoproteins (F and G) on the surface of cells and then measure cell-cell fusion…
C6 ortholog of monkeypox virus [D11L] gene (Unterholzner et al., 2011)	0:3	They did not add monkeypox virus C6 to vaccinia virus
Sheeppox virus SPPV14 can replace F1L in vaccinia virus (Okamoto et al., 2012)	0:3	Sheep pox virus does not cause any significant symptoms in humans, while vaccinia virus does cause mild to moderate symptoms
Recombinant G protein from *Lyssavirus lagos* in attenuated rabies virus strain (Kgaladi et al., 2017)	0:3	They are making chimeras of rabies with a virus that has never been reported to infect humans and that does not have attributes that make it more transmissible than rabies
Ectromelia virus protein ECTV008/OPG012 expressed in Cowpox virus inhibits T cells (Iyer et al., 2022)	0:3	They only investigated the ectromelia virus (ECTV) protein in a virus-free assay of T-cell activation
GoF screen in hepatitis C virus (Grobler et al., 2003)	2:1	HCV strains with higher replication rates are … associated with fibrosing cholestatic hepatitis … experiments that endow high replication rates to this virus should be considered as DURC (A)
E3L-like protein of Orf virus restores IFN resistance to vaccinia virus (Myskiw et al., 2011)	2:1	[Since] E3 proteins could inhibit PKR and IFN antiviral activities, adding them to E3L-deletion vaccinia was an experiment that could be reasonably anticipated to meet DURC (F)…and (A)
Mutations in UL20 of HSV-1 (Charles et al., 2014)	1:2	The creation of replication-enhanced mutants of HSV1, through the introduction of Phe210Ala, would be expected to meet DURC criteria (A)
Orthopoxvirus monkeypox; F3L protein can replace E3 vaccinia protein (Arndt et al., 2015)	1:2	Even though the authors found that replacing E3L from vaccinia with F3L from monkeypox did not result in a virus that inhibits the innate immune system, their expectations when making the chimera seem to have been that it would
MERS-CoV ORF 4a inhibits PKR activation when added to the picornavirus EMCV (Rabouw et al., 2016)	2:1	[S]huffling components from a virus that has a 30% associated mortality (i.e., MERS-CoV) into a virus that can infect humans but which has a very low associated mortality (i.e., ECMV) could result in the chimera with (much) worse properties
Molluscipoxvirus molluscum MC150 and MC160 expressed in vaccinia virus (Biswas et al., 2018)	2:1	Adding genes … known to be innate immune suppressors from MCV to vaccinia virus-vΔA49 could be reasonably anticipated to fall into DURC (A) [or] (F)
gCPXV0030/OPG194 from Cowpox virus into Ratpox virus (Tamošiūnaitė et al., 2020)	1:2	They’ve taken individual genes from a strain that is highly virulent and added them to an avirulent strain to see if it becomes more virulent. That is not reasonably anticipated to result in a virus that is worse than the virulent strain

### Evaluating viral GoF papers for “danger”

While our assessment of whether an experiment constitutes DGoF is based on outcomes, the assessment of proposals for research funding prohibitions or oversight, in practice, occurs at the proposal phase. So, even if the result was not an enhanced pathogen, considerations for research oversight or prohibitions - in practice - take place at the proposal phase, i.e., prior to the actual gain of harmful traits being measured. As such determinations are made in expectation of reasonably anticipated outcomes, prior knowledge about the functions associated with the individual proteins involved in planned experiments is critical.

That said, not all experiments that look as if they will result in DGoF actually do. Sometimes something that looks obvious is not. Research is required because reality is surprising. Without experiments on how pathogens exploit hosts, our knowledge of immunity, particularly innate immunity, would be rudimentary. DGoF research can be necessary, and it is the responsibility of researchers, and the funders who enable it, to see it performed safely such that no recombinant, replicating pathogen is accidently introduced into a host species. Understanding the implications of the biology, principally the sequences involved in microbial pathogenesis, allow researchers, oversight committees, and funders to head off this possibility by selecting appropriate experimental systems and ensuring appropriate, well-designed control measures.

### Limitations

Our screening approach focusing on human-centric dictionary terms and excluding pathogen-centric terms allowed us to focus on manual reviews of just a quarter of the 2100 titles and abstracts. This approach may have missed some DGoF publications (false negatives). Manual review of the residual ∼6K abstracts is subject to all the limitations of human psychology and may have also missed some DGoF publications (more false negatives).

## Conclusion

Our review of the literature in which the GoF term is used and of research which fits the definition of DGoF indicates three important points.

### Most GoF research is not DGoF

Of 20,099 papers mentioning GoF in the title or abstract, only 15 were DGoF that fulfill DURC criteria: one fungal (Na Pombejra et al., 2017), five viral (Grobler et al., 2003; Wang et al., 2011; Sato et al., 2018; Fan et al., 2019; Nakano et al., 2020), and nine bacterial (Uchiyama et al., 2006; Velmurugan et al., 2007; Cardwell and Martinez, 2009; Chang et al., 2011; Hair et al., 2013; Kao et al., 2017; Aghababa et al., 2022; Aspell et al., 2023; Ehtram et al., 2025).

### Most DGoF publications are not referenced with the GoF term

Most researchers doing DGoF on pathogens do not invoke the GoF term. We discuss at least 62 papers in this review, including the supplemental spreadsheet of experiments (Supplementary Table S1), that involve the transfer of SoCs to heterologous microbes, and which demonstrate pathogenic function. Most of these fall into DURC Category A and so meet the definition of DGoF. (Exceptions are the research expressing adhesins in heterologous microbes.) All but twelve of these lack a reference to “gain of function” or “GoF” in the title or abstract.

### Discerning DGoF from GoF is not always a straightforward process

For those who would need to evaluate experimental proposals for DGoF, rubrics that exemplify a “theory of the case” for microbial pathogenesis, including a list of functions for sequences of concern, are valuable. These and the list of the essential DURC criteria are critical for informing a researcher attempting to make these determinations. We included examples of 22 determinations for viral GoF papers involving the transfer of SoCs to heterologous viral species to show the difficulties that can arise in making these determinations, even among experienced professionals. One-third of the articles involved disagreement on whether they qualified as DGoF.

## Methods

A total of 20,347 abstracts on PubMed mentioned the term “Gain of function” in their title or abstracts and were subsequently collected. Abstracts were removed if the PubMed ID was the same and if they had an abstract with fewer than 50 words. Following filtering we were left with 20,099. Dictionaries were created to split abstracts into distinct categories to reduce the number of abstracts that require manual review to identify true DGoF abstracts. Three dictionaries were created including “Human”, “Microbe”, and “Policy”. These dictionaries contain terms (exact strings or regular expressions), commonly associated with humans, microbes, or references to policies. For example, the “Human” dictionary contained terms like *oma, neuro*, tumor*, *autosomal*, *cancer*, or *mitosis*, terms unlikely to be associated with DGoF, see Table 3, below, for example, terms in each dictionary. When abstracts matched only the “Human” terms, they were classified as “GoF_Human” (Figure 1). If they contained terms from the “Microbe” only or both “Microbe” and “Human”, they were classified as “DGoF-other”, then the title was manually reviewed (Figure 1). The manually reviewed titles were then down selected for further classification using the abstract or the entire contents of the paper. The number of instances each term appeared was also recorded for each dictionary. In addition, each abstract was analyzed using a streamlit app using pretrained AI text extraction models including ScispaCy (v0.6.2) NER model “en_ner_bionlp13cg_md” (Neumann et al., 2019), and EasyNER (Ahmed et al., 2023), to extract the organisms mentioned and the disease mentioned in an app developed using Streamlit v1.51.0. The code and complete dictionaries that generated these results are in Dictionaries_Search_GoF_Abstract_Code_Supplemental_File_1. These results assisted our manual classification of the final abstracts to determine if the study was actually DGoF, a policy/commentary/review article, and if the study was an antimicrobial resistance (AMR) study.

**TABLE 3:** Dictionaries and terms for performing preliminary Filter on abstracts containing gain-of-function.

Dictionary	Subset of terms in each dictionary
Human	carcin*, *oma, leuk*, autosomal, autosome, heritable, epigenetic, cancer, tumor*, obesity, neuro*, nervous, TP53, BRCA1, BRCA2, CDK1, CDKN1A, ovary, testis, embryo*
Microbe	Microorganism, microbe*, bacterium, bacteria*, virus, viral, *coccus, pathogenic, fungi, fungal, parasite, infection, infectious, prion*
Policy	Policy, policies

“*” denotes a regular expression (regex) marker for any character before or after the given string, e.g., *oma = lymphoma, carcinoma, melanoma, etc.

## Footnotes

1 Table adapted from report #RR A4907-2 *in preparation*. Zakaria S, Kapoor D, Coringrato E, Sharkey M, Bhangal M. Responsible Innovation in Gain of Function Research and Synthetic Nucleic Acid Technologies. RAND Europe. Cambridge, UK.

## References

[B1] Aghababa H. Ting Y. T. Pilapitiya D. Loh J. M. S. Young P. G. Proft T. (2022). Complement evasion factor (CEF), a novel immune evasion factor of Streptococcus pyogenes. Virulence 13, 225–240. 10.1080/21505594.2022.2027629 35094646 PMC8803112

[B2] Ahmed R. Berntsson P. Skafte A. Rashed S. K. Klang M. Barvesten A. (2023). EasyNER: a customizable easy-to-use pipeline for deep Learning- and Dictionary-based named entity recognition from medical and life science text. arXiv 2304, 07805. Available online at: https://arxiv.org/abs/2304.07805 (Accessed February 9, 2026).

[B3] Ali M. K. Zhen G. Nzungize L. Stojkoska A. Duan X. Li C. (2020). Mycobacterium tuberculosis PE31 (Rv3477) Attenuates host cell apoptosis and promotes recombinant M. smegmatis intracellular survival *via* Up-regulating GTPase guanylate binding Protein-1. Front. Cell Infect. Microbiol. 10, 40. 10.3389/fcimb.2020.00040 32117813 PMC7020884

[B4] Arndt W. D. Cotsmire S. Trainor K. Harrington H. Hauns K. Kibler K. V. (2015). Evasion of the innate immune type I Interferon System by monkeypox virus. J. Virol. 89, 10489–10499. 10.1128/JVI.00304-15 26246580 PMC4580173

[B5] Asmat T. M. Klingbeil K. Jensch I. Burchhardt G. Hammerschmidt S. (2012). Heterologous expression of pneumococcal virulence factor PspC on the surface of Lactococcus lactis confers adhesive properties. Microbiol. Read. 158, 771–780. 10.1099/mic.0.053603-0 22222496

[B6] Aspell T. Khemlani A. H. J. Tsai C. J.-Y. Loh J. M. S. Proft T. (2023). The cell Wall deacetylases Spy1094 and Spy1370 contribute to Streptococcus pyogenes virulence. Microorganisms 11, 305. 10.3390/microorganisms11020305 36838272 PMC9966966

[B7] Balder R. Lipski S. Lazarus J. J. Grose W. Wooten R. M. Hogan R. J. (2010). Identification of Burkholderia mallei and Burkholderia pseudomallei adhesins for human respiratory epithelial cells. BMC Microbiol. 10, 250. 10.1186/1471-2180-10-250 20920184 PMC2955633

[B8] Bartra S. S. Styer K. L. O’Bryant D. M. Nilles M. L. Hinnebusch B. J. Aballay A. (2008). Resistance of Yersinia pestis to complement-dependent killing is mediated by the Ail outer membrane protein. Infect. Immun. 76, 612–622. 10.1128/IAI.01125-07 18025094 PMC2223467

[B9] Biswas S. Smith G. L. Roy E. J. Ward B. Shisler J. L. (2018). A comparison of the effect of molluscum contagiosum virus MC159 and MC160 proteins on vaccinia virus virulence in intranasal and intradermal infection routes. J. Gen. Virol. 99, 246–252. 10.1099/jgv.0.001006 29393023 PMC5884965

[B10] Bliska J. B. Falkow S. (1992). Bacterial resistance to complement killing mediated by the Ail protein of Yersinia enterocolitica. Proc. Natl. Acad. Sci. U. S. A. 89, 3561–3565. 10.1073/pnas.89.8.3561 1565652 PMC48908

[B11] Bossart K. N. Wang L.-F. Flora M. N. Chua K. B. Lam S. K. Eaton B. T. (2002). Membrane fusion tropism and heterotypic functional activities of the Nipah virus and Hendra virus envelope glycoproteins. J. Virol. 76, 11186–11198. 10.1128/jvi.76.22.11186-11198.2002 12388678 PMC136767

[B12] Brennan R. E. Kiss K. Baalman R. Samuel J. E. (2015). Cloning, expression, and characterization of a Coxiella burnetii Cu/Zn Superoxide dismutase. BMC Microbiol. 15, 99. 10.1186/s12866-015-0430-8 25962997 PMC4427992

[B13] Capecchi B. Adu-Bobie J. Di Marcello F. Ciucchi L. Masignani V. Taddei A. (2005). Neisseria meningitidis NadA is a new invasin which promotes bacterial adhesion to and penetration into human epithelial cells. Mol. Microbiol. 55, 687–698. 10.1111/j.1365-2958.2004.04423.x 15660996

[B14] Cardwell M. M. Martinez J. J. (2009). The Sca2 autotransporter protein from Rickettsia conorii is sufficient to mediate adherence to and invasion of cultured mammalian cells. Infect. Immun. 77, 5272–5280. 10.1128/IAI.00201-09 19805531 PMC2786473

[B15] Castiblanco-Valencia M. M. Fraga T. R. Breda L. C. D. Vasconcellos S. A. Figueira C. P. Picardeau M. (2016). Acquisition of negative complement regulators by the saprophyte Leptospira biflexa expressing LigA or LigB confers enhanced survival in human serum. Immunol. Lett. 173, 61–68. 10.1016/j.imlet.2016.03.005 26976804 PMC5437552

[B16] Chang A. Khemlani A. Kang H. Proft T. (2011). Functional analysis of Streptococcus pyogenes nuclease A (SpnA), a novel group A streptococcal virulence factor. Mol. Microbiol. 79, 1629–1642. 10.1111/j.1365-2958.2011.07550.x 21231972

[B17] Charles A.-S. Chouljenko V. N. Jambunathan N. Subramanian R. Mottram P. Kousoulas K. G. (2014). Phenylalanine residues at the carboxyl terminus of the herpes simplex virus 1 UL20 membrane protein regulate cytoplasmic virion envelopment and infectious virus production. J. Virol. 88, 7618–7627. 10.1128/JVI.00657-14 24760889 PMC4054454

[B18] Cohen J. Kaiser J. (2025). NIH suspends alleged “gain-of-function” studies. Science 389, 223–224. 10.1126/science.aea6494 40674472

[B19] Costacurta F. Dodaro A. Bante D. Schöppe H. Sprenger B. Moghadasi S. A. (2023). A comprehensive study of SARS-CoV-2 main protease (Mpro) inhibitor-resistant mutants selected in a VSV-based system. bioRxiv 2023 (22), 558628. 10.1101/2023.09.22.558628 PMC11407635 39259728

[B20] Cui Z. Dang G. Song N. Cui Y. Li Z. Zang X. (2020). Rv3091, an extracellular patatin-like phospholipase in Mycobacterium tuberculosis, prolongs intracellular survival of recombinant Mycolicibacterium smegmatis by mediating phagosomal escape. Front. Microbiol. 11, 2204. 10.3389/fmicb.2020.532371 33042041 PMC7517356

[B21] Cunha L. D. Ribeiro J. M. Fernandes T. D. Massis L. M. Khoo C. A. Moffatt J. H. (2015). Inhibition of inflammasome activation by Coxiella burnetii type IV secretion system effector IcaA. Nat. Commun. 6, 10205. 10.1038/ncomms10205 26687278 PMC4703858

[B22] Del Tordello E. Vacca I. Ram S. Rappuoli R. Serruto D. (2014). Neisseria meningitidis NalP cleaves human complement C3, facilitating degradation of C3b and survival in human serum. Proc. Natl. Acad. Sci. U. S. A. 111, 427–432. 10.1073/pnas.1321556111 24367091 PMC3890809

[B23] Deng W. Yang W. Zeng J. Abdalla A. E. Xie J. (2016). Mycobacterium tuberculosis PPE32 promotes cytokines production and host cell apoptosis through caspase cascade accompanying with enhanced ER stress response. Oncotarget 7, 67347–67359. 10.18632/oncotarget.12030 27634911 PMC5341880

[B24] Deng W. Long Q. Zeng J. Li P. Yang W. Chen X. (2017). Mycobacterium tuberculosis PE_PGRS41 enhances the intracellular survival of M. smegmatis within macrophages *via* blocking innate immunity and inhibition of host defense. Sci. Rep. 7, 46716. 10.1038/srep46716 28440335 PMC5404228

[B25] Deng L. Spencer B. L. Holmes J. A. Mu R. Rego S. Weston T. A. (2019). The Group B Streptococcal surface antigen I/II protein, BspC, interacts with host vimentin to promote adherence to brain endothelium and inflammation during the pathogenesis of meningitis. PLoS Pathog. 15, e1007848. 10.1371/journal.ppat.1007848 31181121 PMC6586375

[B26] Department of Health and Human Services (2017). Framework for guiding funding decisions about proposed research involving enhanced potential pandemic pathogens. Available online at: https://www.phe.gov/s3/dualuse/Documents/P3CO.pdf (Accessed June 7, 2022).

[B27] Ehtram A. Shariq M. Quadir N. Jamal S. Pichipalli M. Zarin S. (2025). Deciphering the functional roles of PE18 and PPE26 proteins in modulating Mycobacterium tuberculosis pathogenesis and immune response. Front. Immunol. 16, 1517822. 10.3389/fimmu.2025.1517822 39949767 PMC11821933

[B28] EOP (2025). Improving the safety and security of biological research. Available online at: https://www.whitehouse.gov/presidential-actions/2025/05/improving-the-safety-and-security-of-biological-research/(Accessed September 17, 2025).

[B29] Fan Y.-C. Liang J.-J. Chen J.-M. Lin J.-W. Chen Y.-Y. Su K.-H. (2019). NS2B/NS3 mutations enhance the infectivity of genotype I Japanese encephalitis virus in amplifying hosts. PLoS Pathog. 15, e1007992. 10.1371/journal.ppat.1007992 31381617 PMC6695206

[B30] Fernandez-Sesma A. Marukian S. Ebersole B. J. Kaminski D. Park M.-S. Yuen T. (2006). Influenza virus evades innate and adaptive immunity *via* the NS1 protein. J. Virol. 80, 6295–6304. 10.1128/JVI.02381-05 16775317 PMC1488970

[B31] Feuerbacher L. A. Hardwidge P. R. (2014). Influence of NleH effector expression, host genetics, and inflammation on Citrobacter rodentium colonization of mice. Microbes Infect. 16, 429–433. 10.1016/j.micinf.2014.02.003 24613200 PMC4040159

[B32] Figueira C. P. Croda J. Choy H. A. Haake D. A. Reis M. G. Ko A. I. (2011). Heterologous expression of pathogen-specific genes ligA and ligB in the saprophyte Leptospira biflexa confers enhanced adhesion to cultured cells and fibronectin. BMC Microbiol. 11, 129. 10.1186/1471-2180-11-129 21658265 PMC3133549

[B33] Fruchart-Gaillard C. Mourier G. Blanchet G. Vera L. Gilles N. Ménez R. (2012). Engineering of three-finger fold toxins creates ligands with original pharmacological profiles for muscarinic and adrenergic receptors. PLoS One 7, e39166. 10.1371/journal.pone.0039166 22720062 PMC3375269

[B34] Fu Y. Phan Q. T. Luo G. Solis N. V. Liu Y. Cormack B. P. (2013). Investigation of the function of Candida albicans Als3 by heterologous expression in Candida glabrata. Infect. Immun. 81, 2528–2535. 10.1128/IAI.00013-13 23630968 PMC3697595

[B35] Gardner C. L. Choi-Nurvitadhi J. Sun C. Bayer A. Hritz J. Ryman K. D. (2013). Natural variation in the heparan sulfate binding domain of the eastern equine encephalitis virus E2 glycoprotein alters interactions with cell surfaces and virulence in mice. J. Virol. 87, 8582–8590. 10.1128/JVI.00937-13 23720725 PMC3719831

[B36] Gemler B. T. Mukherjee C. Howland C. A. Huk D. Shank Z. Harbo L. J. (2022). Function-based classification of hazardous biological sequences: demonstration of a new paradigm for biohazard assessments. Front. Bioeng. Biotechnol. 10, 979497. 10.3389/fbioe.2022.979497 36277394 PMC9585941

[B37] Gill W. P. Harik N. S. Whiddon M. R. Liao R. P. Mittler J. E. Sherman D. R. (2009). A replication clock for Mycobacterium tuberculosis. Nat. Med. 15, 211–214. 10.1038/nm.1915 19182798 PMC2779834

[B38] Gillum D. R. (2025). A possible turning point for research governance in the life sciences. mSphere 10, e0040725. 10.1128/msphere.00407-25 40693791 PMC12379582

[B39] Godbold G. D. Kappell A. D. LeSassier D. S. Treangen T. J. Ternus K. L. (2022). Categorizing sequences of concern by function to better assess mechanisms of microbial pathogenesis. Infect. Immun. 90, e0033421. 10.1128/IAI.00334-21 34780277 PMC9119117

[B40] Gong Z. Yang W. Zhang H. Xiang X. Zeng J. Han S. (2020). Mycobacterium tuberculosis Rv3717 enhances the survival of Mycolicibacterium smegmatis by inhibiting host innate immune and caspase-dependent apoptosis. Infect. Genet. Evol. 84, 104412. 10.1016/j.meegid.2020.104412 32531516

[B41] Grobler J. A. Markel E. J. Fay J. F. Graham D. J. Simcoe A. L. Ludmerer S. W. (2003). Identification of a key determinant of hepatitis C virus cell culture adaptation in domain II of NS3 helicase. J. Biol. Chem. 278, 16741–16746. 10.1074/jbc.M212602200 12615931

[B42] Guimarães V. D. Gabriel J. E. Lefèvre F. Cabanes D. Gruss A. Cossart P. (2005). Internalin-expressing Lactococcus lactis is able to invade small intestine of Guinea pigs and deliver DNA into mammalian epithelial cells. Microbes Infect. 7, 836–844. 10.1016/j.micinf.2005.02.012 15878681

[B43] Guyard C. Battisti J. M. Raffel S. J. Schrumpf M. E. Whitney A. R. Krum J. G. (2006). Relapsing fever spirochaetes produce a serine protease that provides resistance to oxidative stress and killing by neutrophils. Mol. Microbiol. 60, 710–722. 10.1111/j.1365-2958.2006.05122.x 16629672

[B44] Hair P. S. Foley C. K. Krishna N. K. Nyalwidhe J. O. Geoghegan J. A. Foster T. J. (2013). Complement regulator C4BP binds to Staphylococcus aureus surface proteins SdrE and Bbp inhibiting bacterial opsonization and killing. Results Immunol. 3, 114–121. 10.1016/j.rinim.2013.10.004 24600566 PMC3908334

[B45] Heffernan B. J. Thomason B. Herring-Palmer A. Hanna P. (2007). Bacillus anthracis anthrolysin O and three phospholipases C are functionally redundant in a murine model of inhalation anthrax. FEMS Microbiol. Lett. 271, 98–105. 10.1111/j.1574-6968.2007.00713.x 17419764

[B46] Heise T. Dersch P. (2006). Identification of a domain in Yersinia virulence factor YadA that is crucial for extracellular matrix-specific cell adhesion and uptake. Proc. Natl. Acad. Sci. U. S. A. 103, 3375–3380. 10.1073/pnas.0507749103 16488979 PMC1413876

[B47] Hillman R. D. Baktash Y. M. Martinez J. J. (2013). OmpA-mediated rickettsial adherence to and invasion of human endothelial cells is dependent upon interaction with α2β1 integrin. Cell Microbiol. 15, 727–741. 10.1111/cmi.12068 23145974 PMC3610814

[B48] Hirt H. Erlandsen S. L. Dunny G. M. (2000). Heterologous inducible expression of Enterococcus faecalis pCF10 aggregation substance asc10 in Lactococcus lactis and Streptococcus gordonii contributes to cell hydrophobicity and adhesion to fibrin. J. Bacteriol. 182, 2299–2306. 10.1128/JB.182.8.2299-2306.2000 10735875 PMC111281

[B49] Hnátová M. Gbelská Y. Obernauerová M. Subíková V. Subík J. (2003). Cross-resistance to strobilurin fungicides in mitochondrial and nuclear mutants of Saccharomyces cerevisiae. Folia Microbiol. (Praha) 48, 496–500. 10.1007/BF02931331 14533481

[B50] Holmes A. R. McNab R. Millsap K. W. Rohde M. Hammerschmidt S. Mawdsley J. L. (2001). The pavA gene of Streptococcus pneumoniae encodes a fibronectin-binding protein that is essential for virulence. Mol. Microbiol. 41, 1395–1408. 10.1046/j.1365-2958.2001.02610.x 11580843

[B51] Huang X.-Z. Lindler L. E. (2004). The pH 6 antigen is an antiphagocytic factor produced by Yersinia pestis independent of Yersinia outer proteins and capsule antigen. Infect. Immun. 72, 7212–7219. 10.1128/IAI.72.12.7212-7219.2004 15557646 PMC529099

[B52] Hynes W. Sloan M. (2016). “Secreted extracellular virulence factors,” in Streptococcus pyogenes: Basic Biology to Clinical Manifestations. Editors Ferretti J. J. Stevens D. L. Fischetti V. A. (Oklahoma City, OK: The University of Oklahoma Health Sciences Center). Available online at: https://pubmed.ncbi.nlm.nih.gov/26866214/ (Accessed August 13, 2020). 26866208

[B53] Iantomasi R. Sali M. Cascioferro A. Palucci I. Zumbo A. Soldini S. (2012). PE_PGRS30 is required for the full virulence of Mycobacterium tuberculosis. Cell Microbiol. 14, 356–367. 10.1111/j.1462-5822.2011.01721.x 22050772

[B54] Inglesby T. V. Lipsitch M. (2020). Proposed changes to U.S. Policy on potential pandemic pathogen oversight and implementation. mSphere 5, e00990. 10.1128/mSphere.00990-19 31969482 PMC6977183

[B55] Iyer R. F. Edwards D. M. Kolb P. Raué H.-P. Nelson C. A. Epperson M. L. (2022). The secreted protein Cowpox Virus 14 contributes to viral virulence and immune evasion by engaging fc-gamma-receptors. PLoS Pathog. 18, e1010783. 10.1371/journal.ppat.1010783 36121874 PMC9521928

[B130] Jose L. Ramachandran R. Bhagavat R. Gomez R. L. Chandran A. Raghunandanan S. (2016). Hypothetical protein Rv3423.1 of Mycobacterium tuberculosis is a histone acetyltransferase. FEBS J. 283 (2), 265–281. 10.1111/febs.13566 26476134

[B56] Kao W.-C. A. Pětrošová H. Ebady R. Lithgow K. V. Rojas P. Zhang Y. (2017). Identification of Tp0751 (Pallilysin) as a Treponema pallidum Vascular Adhesin by heterologous expression in the lyme disease spirochete. Sci. Rep. 7, 1538. 10.1038/s41598-017-01589-4 28484210 PMC5431505

[B57] Kgaladi J. Faber M. Dietzschold B. Nel L. H. Markotter W. (2017). Pathogenicity and immunogenicity of recombinant rabies viruses expressing the Lagos bat virus matrix and glycoprotein: perspectives for a pan-lyssavirus vaccine. Trop. Med. Infect. Dis. 2, 37. 10.3390/tropicalmed2030037 30270894 PMC6082111

[B58] Kim J.-S. Kim H. K. Cho E. Mun S.-J. Jang S. Jang J. (2022). PE_PGRS38 interaction with HAUSP downregulates antimycobacterial host defense *via* TRAF6. Front. Immunol. 13, 862628. 10.3389/fimmu.2022.862628 35572598 PMC9095961

[B59] Koblentz G. D. Casagrande R. (2023). Beyond gain of function: strengthening oversight of research with potential pandemic pathogens. Pathog. Glob. Health 118, 1–12. 10.1080/20477724.2023.2265627 PMC11221463 37794694

[B60] Krauson A. J. He J. Wimley W. C. (2012). Gain-of-function analogues of the pore-forming peptide melittin selected by orthogonal high-throughput screening. J. Am. Chem. Soc. 134, 12732–12741. 10.1021/ja3042004 22731650 PMC3443472

[B61] Krinos C. High A. S. Rodgers F. G. (1999). Role of the 25 kDa major outer membrane protein of Legionella pneumophila in attachment to U-937 cells and its potential as a virulence factor for chick embryos. J. Appl. Microbiol. 86, 237–244. 10.1046/j.1365-2672.1999.00667.x 10063623

[B62] Kurupati P. Turner C. E. Tziona I. Lawrenson R. A. Alam F. M. Nohadani M. (2010). Chemokine-cleaving Streptococcus pyogenes protease SpyCEP is necessary and sufficient for bacterial dissemination within soft tissues and the respiratory tract. Mol. Microbiol. 76, 1387–1397. 10.1111/j.1365-2958.2010.07065.x 20158613 PMC2904501

[B63] Lafontaine E. R. Balder R. Michel F. Hogan R. J. (2014). Characterization of an autotransporter adhesin protein shared by Burkholderia mallei and Burkholderia pseudomallei. BMC Microbiol. 14, 92. 10.1186/1471-2180-14-92 24731253 PMC4021183

[B64] Leung L. W. Park M.-S. Martinez O. Valmas C. López C. B. Basler C. F. (2011). Ebolavirus VP35 suppresses IFN production from conventional but not plasmacytoid dendritic cells. Immunol. Cell Biol. 89, 792–802. 10.1038/icb.2010.169 21263462 PMC4148147

[B65] Li H. Li Q. Yu Z. Zhou M. Xie J. (2016). Mycobacterium tuberculosis PE13 (Rv1195) manipulates the host cell fate *via* p38-ERK-NF-κB axis and apoptosis. Apoptosis 21, 795–808. 10.1007/s10495-016-1249-y 27147522

[B66] Li F. Feng L. Jin C. Wu X. Fan L. Xiong S. (2018). LpqT improves mycobacteria survival in macrophages by inhibiting TLR2 mediated inflammatory cytokine expression and cell apoptosis. Tuberc. (Edinb) 111, 57–66. 10.1016/j.tube.2018.05.007 30029916

[B67] Li J. Brandalise D. Coste A. T. Sanglard D. Lamoth F. (2024). Exploration of novel mechanisms of azole resistance in Candida auris. Antimicrob. Agents Chemother. 68, e0126524. 10.1128/aac.01265-24 39480072 PMC11619343

[B68] Liesman R. M. Buchholz U. J. Luongo C. L. Yang L. Proia A. D. DeVincenzo J. P. (2014). RSV-encoded NS2 promotes epithelial cell shedding and distal airway obstruction. J. Clin. Invest 124, 2219–2233. 10.1172/JCI72948 24713657 PMC4001550

[B69] Lohberger A. Coste A. T. Sanglard D. (2014). Distinct roles of Candida albicans drug resistance transcription factors TAC1, MRR1, and UPC2 in virulence. Eukaryot. Cell 13, 127–142. 10.1128/EC.00245-13 24243794 PMC3910953

[B70] Long Q. Xiang X. Yin Q. Li S. Yang W. Sun H. (2019). PE_PGRS62 promotes the survival of Mycobacterium smegmatis within macrophages *via* disrupting ER stress-mediated apoptosis. J. Cell Physiol. 234, 19774–19784. 10.1002/jcp.28577 30937925

[B71] Lyon L. M. Doran K. S. Horswill A. R. (2023). Staphylococcus aureus fibronectin-binding proteins contribute to colonization of the female reproductive tract. Infect. Immun. 91, e0046022. 10.1128/iai.00460-22 36511703 PMC9872658

[B72] Manjunath P. Ahmad J. Samal J. Rani A. Sheikh J. A. Zarin S. (2024). Expression of a unique M. tuberculosis DNA MTase Rv1509 in M. smegmatis alters the gene expression pattern and enhances virulence. Front. Microbiol. 15, 1344857. 10.3389/fmicb.2024.1344857 38803374 PMC11129820

[B73] Meng L. Tong J. Wang H. Tao C. Wang Q. Niu C. (2017). PPE38 protein of Mycobacterium tuberculosis inhibits macrophage MHC class I expression and dampens CD8+ T cell responses. Front. Cell Infect. Microbiol. 7, 68. 10.3389/fcimb.2017.00068 28348981 PMC5346565

[B74] Mohanty S. Dal Molin M. Ganguli G. Padhi A. Jena P. Selchow P. (2016). Mycobacterium tuberculosis EsxO (Rv2346c) promotes bacillary survival by inducing oxidative stress mediated genomic instability in macrophages. Tuberc. (Edinb) 96, 44–57. 10.1016/j.tube.2015.11.006 26786654

[B75] Mosser E. M. Rest R. F. (2006). The Bacillus anthracis cholesterol-dependent cytolysin, Anthrolysin O, kills human neutrophils, monocytes and macrophages. BMC Microbiol. 6, 56. 10.1186/1471-2180-6-56 16790055 PMC1550246

[B76] Mu R. Kim B. J. Paco C. Del Rosario Y. Courtney H. S. Doran K. S. (2014). Identification of a group B streptococcal fibronectin binding protein, SfbA, that contributes to invasion of brain endothelium and development of meningitis. Infect. Immun. 82, 2276–2286. 10.1128/IAI.01559-13 24643538 PMC4019170

[B77] Myskiw C. Arsenio J. Hammett C. van Bruggen R. Deschambault Y. Beausoleil N. (2011). Comparative analysis of poxvirus orthologues of the vaccinia virus E3 protein: modulation of protein kinase R activity, cytokine responses, and virus pathogenicity. J. Virol. 85, 12280–12291. 10.1128/JVI.05505-11 21917954 PMC3209343

[B78] Na Pombejra S. Salemi M. Phinney B. S. Gelli A. (2017). The metalloprotease, Mpr1, engages AnnexinA2 to promote the transcytosis of fungal cells across the blood-brain barrier. Front. Cell Infect. Microbiol. 7, 296. 10.3389/fcimb.2017.00296 28713781 PMC5492700

[B79] Nair M. K. M. De Masi L. Yue M. Galván E. M. Chen H. Wang F. (2015). Adhesive properties of YapV and paralogous autotransporter proteins of Yersinia pestis. Infect. Immun. 83, 1809–1819. 10.1128/IAI.00094-15 25690102 PMC4399037

[B80] Nakano Y. Yamamoto K. Ueda M. T. Soper A. Konno Y. Kimura I. (2020). A role for gorilla APOBEC3G in shaping lentivirus evolution including transmission to humans. PLoS Pathog. 16, e1008812. 10.1371/journal.ppat.1008812 32913367 PMC7482973

[B81] Neumann M. King D. Beltagy I. Ammar W. (2019). Fast and robust models for biomedical natural language processing. English, 319–327. 10.18653/v1/W19-5034

[B82] Okamoto T. Campbell S. Mehta N. Thibault J. Colman P. M. Barry M. (2012). Sheeppox virus SPPV14 encodes a Bcl-2-like cell death inhibitor that counters a distinct set of mammalian proapoptotic proteins. J. Virol. 86, 11501–11511. 10.1128/JVI.01115-12 22896610 PMC3486325

[B83] Ortega-Barria E. Pereira M. E. (1991). A novel T. cruzi heparin-binding protein promotes fibroblast adhesion and penetration of engineered bacteria and trypanosomes into mammalian cells. Cell 67, 411–421. 10.1016/0092-8674(91)90192-2 1655283

[B84] Peng X. Luo T. Zhai X. Zhang C. Suo J. Ma P. (2019). PPE11 of Mycobacterium tuberculosis can alter host inflammatory response and trigger cell death. Microb. Pathog. 126, 45–55. 10.1016/j.micpath.2018.10.031 30366125

[B85] Piscatelli H. Kotkar S. A. McBee M. E. Muthupalani S. Schauer D. B. Mandrell R. E. (2011). The EHEC type III effector NleL is an E3 ubiquitin ligase that modulates pedestal formation. PLoS One 6, e19331. 10.1371/journal.pone.0019331 21541301 PMC3082576

[B86] Prasadarao N. V. Lysenko E. Wass C. A. Kim K. S. Weiser J. N. (1999). Opacity-associated protein A contributes to the binding of Haemophilus influenzae to chang epithelial cells. Infect. Immun. 67, 4153–4160. 10.1128/IAI.67.8.4153-4160.1999 10417187 PMC96720

[B87] Primus S. Rocha S. C. Giacani L. Parveen N. (2020). Identification and functional assessment of the first placental adhesin of Treponema pallidum that May play critical role in Congenital Syphilis. Front. Microbiol. 11, 621654. 10.3389/fmicb.2020.621654 33408711 PMC7779807

[B88] Qian J. Hu Y. Zhang X. Chi M. Xu S. Wang H. (2022). Mycobacterium tuberculosis PE_PGRS19 induces pyroptosis through a non-classical Caspase-11/GSDMD pathway in macrophages. Microorganisms 10, 2473. 10.3390/microorganisms10122473 36557726 PMC9785159

[B89] Rabouw H. H. Langereis M. A. Knaap R. C. M. Dalebout T. J. Canton J. Sola I. (2016). Middle East respiratory Coronavirus accessory protein 4a inhibits PKR-Mediated antiviral stress responses. PLoS Pathog. 12, e1005982. 10.1371/journal.ppat.1005982 27783669 PMC5081173

[B90] Rauch S. Costacurta F. Schöppe H. Peng J.-Y. Bante D. Erisoez E. E. (2024). Highly specific SARS-CoV-2 main protease (Mpro) mutations against the clinical antiviral ensitrelvir selected in a safe, VSV-based system. Antivir. Res. 231, 105969. 10.1016/j.antiviral.2024.105969 39053514

[B91] Rego S. Heal T. J. Pidwill G. R. Till M. Robson A. Lamont R. J. (2016). Structural and functional analysis of cell wall-anchored polypeptide adhesin BspA in Streptococcus agalactiae. J. Biol. Chem. 291, 15985–16000. 10.1074/jbc.M116.726562 27311712 PMC4965550

[B92] Reis O. Sousa S. Camejo A. Villiers V. Gouin E. Cossart P. (2010). LapB, a novel Listeria monocytogenes LPXTG surface adhesin, required for entry into eukaryotic cells and virulence. J. Infect. Dis. 202, 551–562. 10.1086/654880 20617901

[B93] Riley S. P. Goh K. C. Hermanas T. M. Cardwell M. M. Chan Y. G. Y. Martinez J. J. (2010). The Rickettsia conorii autotransporter protein Sca1 promotes adherence to nonphagocytic mammalian cells. Infect. Immun. 78, 1895–1904. 10.1128/IAI.01165-09 20176791 PMC2863548

[B94] Riley S. P. Patterson J. L. Nava S. Martinez J. J. (2014). Pathogenic Rickettsia species acquire vitronectin from human serum to promote resistance to complement-mediated killing. Cell Microbiol. 16, 849–861. 10.1111/cmi.12243 24286496 PMC4028375

[B95] Royan S. V. Jones R. M. Koutsouris A. Roxas J. L. Falzari K. Weflen A. W. (2010). Enteropathogenic E. coli non-LEE encoded effectors NleH1 and NleH2 attenuate NF-κB activation. Mol. Microbiol. 78, 1232–1245. 10.1111/j.1365-2958.2010.07400.x 21091507 PMC3325542

[B96] Ruan C. Li J. Niu J. Li P. Huang Y. Li X. (2020). Mycobacterium tuberculosis Rv0426c promotes recombinant mycobacteria intracellular survival *via* manipulating host inflammatory cytokines and suppressing cell apoptosis. Infect. Genet. Evol. 77, 104070. 10.1016/j.meegid.2019.104070 31614213

[B97] Ruiz-Ranwez V. Posadas D. M. Estein S. M. Abdian P. L. Martin F. A. Zorreguieta A. (2013). The BtaF trimeric autotransporter of Brucella suis is involved in attachment to various surfaces, resistance to serum and virulence. PLoS ONE 8, e79770. 10.1371/journal.pone.0079770 24236157 PMC3827427

[B98] Sato K. Misawa N. Takeuchi J. S. Kobayashi T. Izumi T. Aso H. (2018). Experimental adaptive evolution of simian immunodeficiency virus SIVcpz to pandemic human immunodeficiency virus type 1 by using a humanized mouse model. J. Virol. 92, e01905–e01917. 10.1128/JVI.01905-17 29212937 PMC5790958

[B99] Scarselli M. Serruto D. Montanari P. Capecchi B. Adu-Bobie J. Veggi D. (2006). Neisseria meningitidis NhhA is a multifunctional trimeric autotransporter adhesin. Mol. Microbiol. 61, 631–644. 10.1111/j.1365-2958.2006.05261.x 16803596

[B100] Schmidgen T. Kaiser P. O. Ballhorn W. Franz B. Göttig S. Linke D. (2014). Heterologous expression of Bartonella adhesin A in Escherichia coli by exchange of trimeric autotransporter adhesin domains results in enhanced adhesion properties and a pathogenic phenotype. J. Bacteriol. 196, 2155–2165. 10.1128/JB.01461-13 24682330 PMC4054196

[B101] Schubert A. Zakikhany K. Pietrocola G. Meinke A. Speziale P. Eikmanns B. J. (2004). The fibrinogen receptor FbsA promotes adherence of Streptococcus agalactiae to human epithelial cells. Infect. Immun. 72, 6197–6205. 10.1128/IAI.72.11.6197-6205.2004 15501744 PMC523014

[B102] Serruto D. Adu-Bobie J. Scarselli M. Veggi D. Pizza M. Rappuoli R. (2003). Neisseria meningitidis App, a new adhesin with autocatalytic serine protease activity. Mol. Microbiol. 48, 323–334. 10.1046/j.1365-2958.2003.03420.x 12675794

[B103] Sharp J. A. Echague C. G. Hair P. S. Ward M. D. Nyalwidhe J. O. Geoghegan J. A. (2012). Staphylococcus aureus surface protein SdrE binds complement regulator factor H as an immune evasion tactic. PLoS One 7, e38407. 10.1371/journal.pone.0038407 22675461 PMC3364985

[B104] Sheng X. You Q. Zhu H. Chang Z. Li Q. Wang H. (2017). Bacterial effector NleL promotes enterohemorrhagic E. Coli-induced attaching and effacing lesions by ubiquitylating and inactivating JNK. PLoS Pathog. 13, e1006534. 10.1371/journal.ppat.1006534 28753655 PMC5549993

[B105] Sparks I. L. Derbyshire K. M. Jacobs W. R. Morita Y. S. (2023). Mycobacterium smegmatis: the Vanguard of Mycobacterial Research. J. Bacteriol. 205, e0033722. 10.1128/jb.00337-22 36598232 PMC9879119

[B106] Srivastava S. Battu M. B. Khan M. Z. Nandicoori V. K. Mukhopadhyay S. (2019). Mycobacterium tuberculosis PPE2 protein interacts with p67phox and inhibits reactive oxygen Species production. J. Immunol. 203, 1218–1229. 10.4049/jimmunol.1801143 31375544

[B107] St Geme J. W. Falkow S. Barenkamp S. J. (1993). High-molecular-weight proteins of nontypable Haemophilus influenzae mediate attachment to human epithelial cells. Proc. Natl. Acad. Sci. U. S. A. 90, 2875–2879. 10.1073/pnas.90.7.2875 8464902 PMC46199

[B108] Stutzmann Meier P. Entenza J. M. Vaudaux P. Francioli P. Glauser M. P. Moreillon P. (2001). Study of Staphylococcus aureus pathogenic genes by transfer and expression in the less virulent organism Streptococcus gordonii. Infect. Immun. 69, 657–664. 10.1128/IAI.69.2.657-664.2001 11159952 PMC97936

[B109] Su H. Zhu S. Zhu L. Huang W. Wang H. Zhang Z. (2016). Recombinant lipoprotein Rv1016c derived from Mycobacterium tuberculosis is a TLR-2 ligand that induces macrophages apoptosis and inhibits MHC II antigen processing. Front. Cell Infect. Microbiol. 6, 147. 10.3389/fcimb.2016.00147 27917375 PMC5114242

[B110] Tamošiūnaitė A. Weber S. Schippers T. Franke A. Xu Z. Jenckel M. (2020). What a difference a gene makes: identification of virulence factors of cowpox virus. J. Virol. 94, e01625-19. 10.1128/JVI.01625-19 31645446 PMC6955266

[B111] Thi E. P. Hong C. J. H. Sanghera G. Reiner N. E. (2013). Identification of the Mycobacterium tuberculosis protein PE-PGRS62 as a novel effector that functions to block phagosome maturation and inhibit iNOS expression. Cell. Microbiol. 15, 795–808. 10.1111/cmi.12073 23167250

[B112] Tiwari B. M. Kannan N. Vemu L. Raghunand T. R. (2012). The Mycobacterium tuberculosis PE proteins Rv0285 and Rv1386 modulate innate immunity and mediate bacillary survival in macrophages. PLoS ONE 7, e51686. 10.1371/journal.pone.0051686 23284742 PMC3524191

[B113] Turner D. P. J. Marietou A. G. Johnston L. Ho K. K. L. Rogers A. J. Wooldridge K. G. (2006). Characterization of MspA, an immunogenic autotransporter protein that mediates adhesion to epithelial and endothelial cells in Neisseria meningitidis. Infect. Immun. 74, 2957–2964. 10.1128/IAI.74.5.2957-2964.2006 16622234 PMC1459726

[B114] Uchiyama T. Kawano H. Kusuhara Y. (2006). The major outer membrane protein rOmpB of spotted fever group rickettsiae functions in the rickettsial adherence to and invasion of Vero cells. Microbes Infect. 8, 801–809. 10.1016/j.micinf.2005.10.003 16500128

[B115] Ullah H. Shi X. Taj A. Cheng L. Yan Q. Sha S. (2024). Mycobacterium tuberculosis PE_PGRS38 enhances intracellular survival of mycobacteria by inhibiting TLR4/NF-κB-Dependent inflammation and apoptosis of the host. Biol. (Basel) 13, 313. 10.3390/biology13050313 38785795 PMC11118070

[B116] United States Government (2012). United States government Policy for oversight of life sciences dual use research of concern. Available online at: https://www.phe.gov/s3/dualuse/Documents/us-policy-durc-032812.pdf (Accessed June 7, 2022).

[B117] United States Government (2014). United States government Policy for institutional oversight of life sciences dual use research of concern. Available online at: https://www.phe.gov/s3/dualuse/Documents/durc-policy.pdf (Accessed June 7, 2022).

[B118] Unterholzner L. Sumner R. P. Baran M. Ren H. Mansur D. S. Bourke N. M. (2011). Vaccinia virus protein C6 is a virulence factor that binds TBK-1 adaptor proteins and inhibits activation of IRF3 and IRF7. PLoS Pathog. 7, e1002247. 10.1371/journal.ppat.1002247 21931555 PMC3169548

[B119] Velmurugan K. Chen B. Miller J. L. Azogue S. Gurses S. Hsu T. (2007). Mycobacterium tuberculosis nuoG is a virulence gene that inhibits apoptosis of infected host cells. PLoS Pathog. 3, e110. 10.1371/journal.ppat.0030110 17658950 PMC1924871

[B120] Verhoef K. Berkhout B. (1999). A second-site mutation that restores replication of a Tat-defective human immunodeficiency virus. J. Virol. 73, 2781–2789. 10.1128/JVI.73.4.2781-2789.1999 10074125 PMC104035

[B121] Wang W. Donnelly B. Bondoc A. Mohanty S. K. McNeal M. Ward R. (2011). The rhesus rotavirus gene encoding VP4 is a major determinant in the pathogenesis of biliary atresia in newborn mice. J. Virol. 85, 9069–9077. 10.1128/JVI.02436-10 21697466 PMC3165802

[B122] Wang L. Zuo M. Chen H. Liu S. Wu X. Cui Z. (2017). Mycobacterium tuberculosis Lipoprotein MPT83 induces apoptosis of infected macrophages by activating the TLR2/p38/COX-2 signaling pathway. J. Immunol. 198, 4772–4780. 10.4049/jimmunol.1700030 28507027

[B123] Wei Z. Schnupf P. Poussin M. A. Zenewicz L. A. Shen H. Goldfine H. (2005). Characterization of Listeria monocytogenes expressing anthrolysin O and phosphatidylinositol-specific phospholipase C from Bacillus anthracis. Infect. Immun. 73, 6639–6646. 10.1128/IAI.73.10.6639-6646.2005 16177340 PMC1230906

[B124] Xia A. Li X. Quan J. Chen X. Xu Z. Jiao X. (2021). Mycobacterium tuberculosis Rv0927c inhibits NF-κB pathway by downregulating the phosphorylation level of IκBα and enhances mycobacterial survival. Front. Immunol. 12, 721370. 10.3389/fimmu.2021.721370 34531869 PMC8438533

[B125] Yan S. Zhen J. Li Y. Zhang C. Stojkoska A. Lambert N. (2019). Mce-associated protein Rv0177 alters the cell wall structure of Mycobacterium smegmatis and promotes macrophage apoptosis *via* regulating the cytokines. Int. Immunopharmacol. 66, 205–214. 10.1016/j.intimp.2018.11.013 30472521

[B126] Yang G. Luo T. Sun C. Yuan J. Peng X. Zhang C. (2017a). PPE27 in Mycobacterium smegmatis enhances mycobacterial survival and manipulates cytokine secretion in mouse macrophages. J. Interferon Cytokine Res. 37, 421–431. 10.1089/jir.2016.0126 28829246

[B127] Yang W. Deng W. Zeng J. Ren S. Ali M. K. Gu Y. (2017b). Mycobacterium tuberculosis PE_PGRS18 enhances the intracellular survival of M. smegmatis *via* altering host macrophage cytokine profiling and attenuating the cell apoptosis. Apoptosis 22, 502–509. 10.1007/s10495-016-1336-0 27987050

[B128] Zenewicz L. A. Wei Z. Goldfine H. Shen H. (2005). Phosphatidylinositol-specific phospholipase C of Bacillus anthracis down-modulates the immune response. J. Immunol. 174, 8011–8016. 10.4049/jimmunol.174.12.8011 15944308

[B129] Zhang P. Chomel B. B. Schau M. K. Goo J. S. Droz S. Kelminson K. L. (2004). A family of variably expressed outer-membrane proteins (Vomp) mediates adhesion and autoaggregation in Bartonella quintana. Proc. Natl. Acad. Sci. U. S. A. 101, 13630–13635. 10.1073/pnas.0405284101 15347808 PMC518805

