# Supplementary material for: Discerning dangerous gain of function: most gain of function (GoF) research does not involve infectious microbes
Source: Front Bioeng Biotechnol. 2026 Jun 19;14:1818657. doi: 10.3389/fbioe.2026.1818657 (PMC13327987; doi:10.3389/fbioe.2026.1818657)
Supplement: Supplementary file 2 [file DataSheet1.pdf]

Full Evaluator Comments on Potential DGoF Viral Papers

Publications which three evaluators unanimously agreed were dangerous gain of function

Influenza virus NS1 transferred to Newcastle Disease Virus,(Fernandez-Sesma et al., 2006)

Evaluator 1: **DGoF**. The introduction of influenza NS1 into Newcastle Disease virus (NCDV), after the demonstration that NS1 inhibits dendritic cell maturation through the inhibition of multiple innate immune modulators, should have been anticipated by the researchers as posing a significant likelihood of enhancing pathogenesis. The researchers don't mention biosafety in the article, and they report using the B1 Hitchner avian vaccine strain of NCDV. Although an attenuated strain, the virus is able to infect the human dendritic cells used in the experiment. The addition of a strong immunomodulator like NS1 could totally change the pathological profile of infection with that attenuated virus. Based on the results in this paper it should have been clear to the researchers that the experimental approach would result in a virus with enhancements to DURC categories (F), susceptibility of humans to infection and (A), enhanced harmful consequences.

Evaluator 2 & **3**: **DGoF**

VP35 from Ebolavirus expressed in Newcastle Disease Virus,(Leung et al., 2011)

Evaluator 1: **DGoF**. The role of VP35 in subverting innate immunity during infection had been established through loss of function studies. So, adding VP35 from Ebola virus to either NCDV or herpes simplex virus (HSV) virus is reasonably anticipated to meet DURC category (F), susceptibility of humans to infection with these viruses and also (A), reasonably anticipated to enhance the harmful consequences of the agent.

Evaluator 2 & **3**: **DGoF**

E3L-like protein from Orf virus restores IFN resistance to vaccinia virus,(Myskiw et al., 2011)

Evaluator 1: **DGoF**. Once the researchers had determined that the myxoma and swinepox virus E3 proteins could inhibit PKR and IFN antiviral activities, adding them to E3L-deletion vaccinia was an experiment that could be reasonably anticipated to meet DURC (F), enhancements to the susceptibility of humans to the agent, and (A), enhanced harmful consequences

Evaluator 2 & **3**: **DGoF**

VP4 of Rhesus rotavirus,(Wang et al., 2011)

Evaluator 1: **DGoF**. The transfer of genes from rhesus rotavirus (RRV), which causes disease in humans, to the TUCH strain of rotavirus – which is not associated with human disease – in a manner that increases the pathogenicity of TUCH in an animal model of infection seems to meet DURC criteria (A), enhancing the harmful consequences from an infectious agent. Whether this is anticipated to result in human-human transmission that is worse than the already endemic rotavirus should be the topic of the discussion about whether this experiment could result in significant societal consequences.

Evaluator 2: **DGoF**. The mutation "enhances pathogenicity" and would enhance "the harmful consequences of the agent". The TUCH mutant had the same viral load and higher mortality rate than the RRV wt, so one could argue that this enhances pathogenicity over wild type RRV (Table 2).

Evaluator 3: **DGoF**

Alphavirus eastern/EEV and the E2 glycoprotein,(Gardner et al., 2013)

Evaluator 1: **DGoF**. In an earlier paper, Ryman et al., 2007, the authors identified some amino acid substitutions that are associated with higher mortality in older mice. That was an unanticipated experimental result that the authors note when discussing the data from Table 3. Using the same approach with another virus indicates that they should have reasonably anticipated that - through these substitutions intended to modify the interaction with heparan sulfate - their anticipated results would fall under DURC (A), enhancing the harmful consequences of the eastern equine encephalitis virus, which already has significant pathogenesis.

Evaluator 2: **DGoF**. I think we should differentiate point mutations from experiments like those in which they are swapping genes. I think the latter is much worse. FWIW, I don't think intent should be part of our evaluation here — we are just evaluating whether or not the experiment itself is bad. For this one, the double mutant decreases average survival time in mice by quite a bit (Table 3) and increases infectivity and PFUs in cell culture. So this is DURC (A), potential "enhance harmful consequences" and possibly even alter tropism.
Evaluator 3: **DGoF**. I agree about point mutations vs. swapping genes or creating chimeric viruses. The probability that nature could produce a similar mutation plays a role in risk here. These single amino acid polymorphisms could absolutely arise on their own if they provided the virus a significant increase in fitness, likely increasing the danger associated with viral infection. Swapping entire genes or genome fragments between viruses with different host ranges is in a different class altogether and is much more likely to happen in a lab.

NS2 from RSV expressed in parainfluenza virus 3,(Liesman et al., 2014)
Evaluator 1: **DGoF**. The scientists characterize the role of the respiratory syncytial virus (RSV) NS2 protein in damaging airway epithelium and then add it to another respiratory virus, parainfluenza virus (PIV), to see if it would make PIV more harmful to airway epithelia. The experimental aim satisfies DURC criteria (A), enhancing the harmful consequences of the agent.
Evaluator 2: **DGoF**. The NS2 protein, when expressed in PIV, led to increased occlusion of airways (Figure 8C) compared to the wild type, which suggests an enhanced virus..
Evaluator 3: DGoF. Definitively DGoF based on the data presented.

Adapting SIV to pandemic HIV-1 using humanized mouse model,(Sato et al., 2018)
Evaluator 1: **DGoF**. The characterization of mutations in the simian immunodeficiency virus (SIV) strain SIVcpzPtt MB897 that are associated with replication in humanized mice isn't problematic. But the introduction of mutations from human immunodeficiency virus (HIV) strain M to determine if they expand the host range and replication efficiency seem to meet DURC criteria (A), enhancing the harmful consequences, and (E), altering the host range or tropism of the agent. Although this strain still doesn't replicate as well as pandemic strains of HIV, the deleterious effects of having a new HIV strain – which may not be susceptible to current therapeutic regime, for instance – would be reasonably anticipated to have significant societal consequences.
Evaluator 2: **DGoF**. It is interesting that the G413R mutational gain of function is unique to the MB897, but the simple fact that the researchers are creating mutants that could cause non-pandemic strains to become pandemic meets the criteria.
Evaluator 3: **DGoF**.

NS2B/NS3 mutations enhance the infectivity of Japanese encephalitis virus,(Fan et al., 2019)
Evaluator 1: **DGoF**. The substitution of elements from a low replication GIII strain of Japanese encephalitic virus (JEV) with elements from the high replication GI strain and the introduction of mutations that further increase the replication efficiency of the GII/GI chimera seems to meet DURC criteria (A), enhancing the harmful consequences of the agent. Additionally, they lead the article by explaining that the GI strain has replaced the GIII strain in the wild in Asia. In carrying out these experiments, they risk creating a chimera that could outcompete GI in the wild, which would have significant societal consequences. It would have been safer to do this experiment with an attenuated version of each strain and to look at the impact on replication kinetics from these experimental manipulations using such viruses that would be each significantly disadvantaged in comparison to the circulating strains.
Evaluator 2: **DGoF**.
Evaluator 3: **DGoF**.

Role of gorilla APOBEC3G in shaping lentivirus evolution including human transmission,(Nakano et al., 2020)
Evaluator 1: **DGoF**. They produced a SIVcpzPtt virus with a mutation in Vif, and identified that the mutation lifts the constraints that prevent replication of a chimpanzee lentivirus in gorilla and human cells. The generation of a mutant SIVcpzPtt that replicated in human cell lines meets the (E) criteria of altering the host range or tropism of this virus in a manner that could cause significant societal consequences.
Evaluator 2: **DGoF**. The fact that the researchers reveal the specific mutant that overcomes host APOBEC3 neutralization of the virus raises it to the level of DGoF
Evaluator 3: **DGoF**.

Publications which three evaluators unanimously agreed were not dangerous gain of function

Tat variants in HIV-1,(Verhoef and Berkhout, 1999)
Evaluator 1: Not DGoF. The identification of Y47N as increasing Tat's potentiation of reverse transcription by 200% but this does not actually benefit viral replication as shown in Fig. 7. Even if it were found to have enhanced replication in the lab, it's not clear that this would lead to greater pathogenesis. However, if Fig 7 showed a significant replicative advantage to Y47N LAI (HIV) virus, then I think that a conservative assessment would deem it to be DGoF. In the discussion, they mention that maybe Tat enhancement by this mutation is killing cells prior to their being able to produce more LAI (HIV) than would otherwise have been produced. This could lead to changes in pathogenesis, and I guess animal infection investigations could clarify.
Evaluator 2 & **3**: Not DGoF

Henipaviruses Glycoprotein shuffling,(Bossart et al., 2002)
Evaluator 1: Not DGoF. They use vaccinia virus as a transduction mechanism to get expression of henipavirus envelope glycoproteins (F and G) on the surface of cells and then measure cell-cell fusion (syncytia formation).
Evaluator 2 & **3**: Not DGoF

C6 ortholog of monkeypox virus [D11L] gene,(Unterholzner et al., 2011)
Evaluator 1: Not DGoF. They didn't add monkeypox virus C6 to vaccinia virus, they merely transfected an expression vector for that protein into cells and looked at the inhibition of poly(dA-dT)-induced IRF3 transactivation – finding that it inhibited the pathway at the level of TBK1 and IKKε.
Evaluator 2 & **3**: Not DGoF

Sheeppox virus SPPV14 can replace F1L in vaccinia virus,(Okamoto et al., 2012)
Evaluator 1: Based on the available data, this is not DGoF. They removed F1L from vaccinia and replaced it with SPPV14, from sheep pox virus. Sheep pox virus does not cause any significant symptoms in humans, while vaccinia virus does cause mild to moderate symptoms. It is possible that some combinations of shuffling of proteins between these huge viruses could result in a virus that is much worse than either of the originals. One could imagine that protein X from sheep pox virus might have bad effects on the host if not for an interaction with protein Y from sheep pox virus, and when introduced into vaccinia the interaction with vaccinia protein Y isn't as strong and now protein X is able to cause harm to the host. It's hard to say definitively. But, in the absence of more published information about the proteins that the researchers are exchanging between these viruses it is not possible to categorize this within the DURC categories.
Evaluator 2: Also leaning toward not DGoF. Fig 3A shows that SPPV14 can slightly increase apoptosis protection relative to M11L, but this is based just on expression of that protein by itself.
Evaluator 3: Not DGoF. They are swapping genes between viruses that appear to have equivalent function resulting in viruses that are more or less equipotent. They are verifying the function of the related gene products. But they are not attempting to add novel functionality to the viruses. There is

risk, and it would be better if the authors found a way to assess the results in a pseudoviral or other attenuated system.

Recombinant G protein from Lyssavirus lagos in attenuated rabies virus strain,(Kgaladi et al., 2017)
Evaluator 1: Not DGoF. They are making chimeras of rabies with a virus that has never been reported to infect humans and that does not have attributes that make it more transmissible than rabies. Before carrying out these experiments it should be clear to the researchers that there is absolutely no evidence that Lagos bat virus is transmissible through the air. As long as there is no reason to believe that Lagos bat virus has no traits that could render rabies enhanced in the manner encompassed by the DURC categories, this is not - a priori - DGoF research.
Evaluator 2: Not DGoF. There is no enhancement.
Evaluator 3: Not DGoF. The properties of the chimeric viruses do not seem to significantly increase transmissibility or dissemination and the chimeras seem to be very immunogenic.

Ectromelia virus protein ECTV008/OPG012 expressed in Cowpox virus inhibits T cells,(Iyer et al., 2022)
Evaluator 1: Not DGoF. They only investigated the ectromelia virus (ECTV) protein in a virus-free assay of T-cell activation
Evaluator 2: Not DGoF.
Evaluator 3: Not DGoF. If their conclusions are correct they could have pivoted to DGoF research, but based on the results in the paper, they did not generate anything that could be construed as DGoF.

Publications where three evaluators were split on whether dangerous gain of function was demonstrated

GoF screen in hepatitis C virus,(Grobler et al., 2003)
Evaluator 1: **DGoF**. The expansion of the "host range" from chimpanzees to human liver cells isn't associated with receptor binding, but rather with replication kinetics. I expect that the wild-type virus they isolated from chimps was already able to replicate in humans, but that the restriction was because these are cultured human cells not a live person. Potentially the double mutant identified would be worse in humans. Also, there is literature describing more severe disease associated with fast-replicating variants of hepatitis C virus (HCV) in immunocompromised patients and more severe pathogenesis. Since there is reason to believe that HCV strains with higher replication rates are generally associated with fibrosing cholestatic hepatitis, it seems that experiments that endow high replication rates to this virus should be considered as DURC (A), enhancing the harmful consequences of the disease, even when the higher replication rate is only measured in cell culture.
Evaluator 2: Not DGoF. One could argue that the "host range" is expanded, but does expanding to a specific cell line really count? I think the potential for enhancing harmful consequences would probably be more due to being able to produce more of it (which is not one of the criteria as far as I can tell), instead of enhancing pathogenicity or transmissibility.
Evaluator 3: **DGoF**. The difference between in vivo and in vitro is academic in some respects – this virus was modified to have expanded host range in a different cell type/organism. I agree with the above points regarding higher replication rates. Given a lack of extensive testing of these mutations across a wide range of organisms and cell types, any conclusions regarding the gain of function being restricted to an *in vitro* environment would be speculative.

E3L-like protein from Orf virus restores IFN resistance to vaccinia virus,(Myskiw et al., 2011)
Evaluator 1: **DGoF**. Once the researchers had determined that the myxoma and swinepox virus E3 proteins could inhibit PKR and IFN antiviral activities, adding them to E3L-deletion vaccinia was an experiment that could be reasonably anticipated to meet DURC (F), enhancements to the susceptibility of humans to the agent, and (A), enhanced harmful consequences

Evaluator 2: If the outcome of the experiment does not result in a "worse" virus then it is not DGoF. I agree that the experiment could have resulted in a worse virus, but Table 1 shows that replacing vaccinia E3L with E3L from orthologs did not restore pathogenicity like vaccinia E3L. This is an important distinction to note: Is the yes/no based on whether the experiment could lead to a worse virus or if it actually does lead to a worse virus *in vivo*?
Evaluator 3: This is probably mild **DGoF** given the finding that the results did not carry over into the *in vivo* system.

Mutations in UL20 of HSV-1,(Charles et al., 2014)
Evaluator 1: **DGoF**. The creation of replication-enhanced mutants of HSV1, through the introduction of Phe210Ala, would be expected to meet DURC criteria (A), enhancing the harmful consequences from an infectious agent and would do so in a manner that could cause significant societal consequences – especially given the wide endemicity of the virus in the human population and the relative difficulty of eradicating infectious with the wild-type strains. In thinking about the pathology of HSV1 infection, i.e., the growth of sores on the skin prior to the immune system reigning in the infection, my assumption is that higher replication rates would correlate with larger sores. Supporting this assessment is that peripheral serotonin levels enhance HSV1 replication and lead to more severe disease outcomes in a rabbit model of HSV corneal disease.
Evaluator 2: Not DGoF. The single log increase is only at the high MOI/cell (Figure 5), and increased production in Vero cells does not necessarily equate to increased pathogenicity or transmissibility. If we want to include mutants that increase production titer as gain of function mutants, then I would lean toward yes, but I don't think these fit the GoF criteria of "enhancing its pathogenicity or increasing its transmissibility." If the authors had shown that the mutant increased pathogenicity in a mouse model or even increased viral load in a mouse, not just *in vitro*, I would say yes, but just with the evidence in this paper, I don't think this one rises to the DGoF level.
Evaluator 3: Not DGoF. I am skeptical the mutation would lead to substantial GoF in other biological contexts. This mutation would not have been a difficult one for evolution to select for on its own if it were as simple as increasing the replication rate without otherwise impacting replication or transmission. This point is further made in the discussion section as they speculate on the potential reasons for the observed differences in different cell lines. Ultimately, the paper does not provide data to make the point that there is significantly enhanced pathogenicity or transmissibility. While it does appear that the replication rate is enhanced at the higher MOI in Figure 5, the subsequent figures do not provide data that suggest the transmissibility of the virus or the severity of the infection (as shown by the plaque morphology and results) is significantly different.

Orthopoxvirus monkeypox; F3L protein can replace E3 vaccinia protein,(Arndt et al., 2015)
Evaluator 1: **DGoF**, or at least would have been when the experiment was proposed. Even though the authors found that replacing E3L from vaccinia with F3L from monkeypox did not result in a virus that inhibits the innate immune system, their expectations when making the chimera seem to have been that it would. So, the scientists should have a reasonable expectation that the changes would meet DURC (A), enhance the harmful consequences of the agent or (F) enhancing the susceptibility of the human host population.

Evaluator 2: Not DGoF. The swap of the monkeypox F3L with vaccinia E3L does not make the vaccinia virus "enhanced". This (again) gets to a difference of whether or not the experiment could result in enhancement compared to whether it actually did.
Evaluator 3: Not DGoF given that the data did not support any gain of function since the virus already contained the functionality elsewhere. I see the points Evaluator 1 makes about the author’s intent and hypothesis when the experiment was proposed, but that is somewhat of a slippery slope. If authors had no reason to suspect potential DGoF but then discovered there was, that would not change the fact that

a DGoF study took place. I am not sure then how fair it is to impose that line of reasoning in the other direction.

MERS-CoV ORF 4a inhibits PKR activation when added to the picornavirus EMCV,(Rabouw et al., 2016)
Evaluator 1: **DGoF**. While it isn't clear that ECMV with the Middle East respiratory syndrome coronavirus (MERS-CoV) P4 protein would be clinically worse than ECMV in the manner than MERS-CoV is clinically worse than ECMV, shuffling components from a virus that has a 30% associated mortality (i.e., MERS-CoV) into a virus that can infect humans but which has a very low associated mortality (i.e., ECMV) could result in the chimera with (much) worse properties. The authors should have known that this was reasonably anticipated to meet the DURC categories of (A), enhancing the harmful consequences of the agent, through accomplishing (F), enhancing the susceptibility of the human host population.
Evaluator 2: Not DGoF. The authors showed that expression of P4a from MERS-CoV in the EMCV virus did not enhance the virus (a different mutation did, Fig 5).
Evaluator 3: **DGoF**. They engineered a chimeric virus to have an inhibitory effect on cell defense strategies using a select agent virus. And then they showed increased replication efficiency.

Molluscipoxvirus molluscum MC150 and MC160 expressed in vaccinia virus,(Biswas et al., 2018)
Evaluator 1: **DGoF**. Adding genes that are known to be innate immune suppressors from MCV to vaccinia virus-vΔA49 could be reasonably anticipated to fall into DURC (A), enhancing the harmful consequences of the agent, by (F), enhancing the susceptibility of the human host population.
Evaluator 2: Not DGoF. The vΔA49rev is more virulent that either of the 2 MCV mutants, so neither lead to enhancement.
Evaluator 3: This is a very low risk case of **DGoF**. They have increased susceptibility of host populations but have done it with a series of highly attenuated viruses that pose a rather low risk outside of a laboratory environment.

gCPXV0030/OPG194 from Cowpox virus into Ratpox virus,(Tamošiūnaitė et al., 2020)
Evaluator 1: Not DGoF. They've taken individual genes from a strain that is highly virulent and added them to an avirulent strain to see if it becomes more virulent. That isn't reasonably anticipated to result in a virus that is worse than the virulent strain.
Evaluator 2: **DGoF**. Genes from the virulent strain, when put in an avirulent background, made the previously avirulent strain virulent.
Evaluator 3: Not DGoF. If their conclusions are correct they could have committed DGoF research pretty readily, but based on the results in the paper, they did not generate anything that could be construed as DGoF.
